# Understanding bacterial biofilms: From definition to treatment strategies

**DOI:** 10.3389/fcimb.2023.1137947

**Published:** 2023-04-06

**Authors:** Ailing Zhao, Jiazheng Sun, Yipin Liu

**Affiliations:** ^1^ Department of Gastroenterology, Yantai Affiliated Hospital of Binzhou Medical University, Yantai, Shandong, China; ^2^ Department of Vasculocardiology, Jinzhou Medical University, Jinzhou, Liaoning, China

**Keywords:** bacterial biofilms, infection, antibiotic resistance, biofilm detection, treatment

## Abstract

Bacterial biofilms are complex microbial communities encased in extracellular polymeric substances. Their formation is a multi-step process. Biofilms are a significant problem in treating bacterial infections and are one of the main reasons for the persistence of infections. They can exhibit increased resistance to classical antibiotics and cause disease through device-related and non-device (tissue) -associated infections, posing a severe threat to global health issues. Therefore, early detection and search for new and alternative treatments are essential for treating and suppressing biofilm-associated infections. In this paper, we systematically reviewed the formation of bacterial biofilms, associated infections, detection methods, and potential treatment strategies, aiming to provide researchers with the latest progress in the detection and treatment of bacterial biofilms.

## 1 Introduction

Bacterial biofilms have become an essential contributor to global health problems due to antibiotic resistance, the host’s immune defense system, and other external pressures. Biofilms are commonly found on the surface of hospital instruments and body tissue, in industry, food processing units, and natural environments (Schulze et al., 2021). Almost all bacteria can form biofilms.

Bacterial biofilms are usually defined as fixed microbial communities encased in extracellular polymeric substances (EPS). It is characterized by changes in the irreversible adhesion of microbial cells to surfaces or substrates or each other, embedded in EPS, and exhibiting specific phenotypes in terms of gene transcription and growth rates. A bacterial biofilm is composed of a single microorganism or a mixture of bacteria, fungi, archaea, protozoa, and yeasts. It has a channel structure that controls the release of gases, nutrients, and antimicrobials.

Free-floating bacterial cells can also aggregate to form biofilms, which exhibit similar characteristics to medical device-related biofilms (Hall-Stoodley et al., 2012). With the improvement of medical technology, the widespread use of medical devices, and the pursuit of high quality of life for patients, medical device-associated biofilms pose a severe threat to the life and health of patients. Microorganisms can adhere to almost all medical devices and cause medical device-associated biofilm infections. Device-associated infections usually occur during treatment, where some microorganisms originate from the host. When these microorganisms attach and colonize the surface of a medical device, they can form a biological container. The pathogenesis of medical device-associated infections is related to microorganisms in complex communities that adhere to and grow on device surfaces. Medical device-related biofilms can consist of single or multiple species, depending primarily on the type of device and the time it is left in the patient’s body. In most cases, device-associated infections are associated with biofilm formation on device surfaces. When floating bacteria come into contact with the surface of a medical device, they secrete polymers that create a three-dimensional matrix, which eventually sticks to the surface of the device, forming a biofilm structure. When the biofilm on the surface of implanted medical devices reaches a critical level, it can induce an inflammatory response in the host and may even cause implant failure. The most common microorganisms for medical device-associated infections are *Staphylococcus aureus* and *Staphylococcus epidermidis*. Multi-resistant gram-negative bacteria (e.g., *Escherichia coli*, *Klebsiella pneumoniae*, *Pseudomonas aeruginosa*, and *Acinetobacter baumannii*) can also cause medical device-associated infections in complex hospital settings (Niveditha et al., 2012). In addition, microorganisms can adhere to various tissue surfaces in the body (e.g., skin, connective tissue, intestinal mucosa, vascular endothelium, oral cavity, airway, bone tissue, and vagina), which in turn can cause non-device (tissue) -associated biofilm infections and lead to various diseases (Wi and Patel, 2018). When microorganisms in the oral cavity attach to enamel, dentin, and mucosal epithelial tissues, they can form a dental plaque, which is influenced by the nature of the attachment surface, the intraoral environment, and the state of oral health.

When stimulated by the harsh environment, the exopolysaccharides, fibrins and lipoproteins secreted by bacteria adhere to the surface of inert objects and form microbial substances. EPS is conducive to bacterial adhesion and promotes the formation of a biofilm matrix composed of extracellular polysaccharides, exogenous DNA, proteins, and lipids (Schilcher and Horswill, 2020; Chiba et al., 2022). The EPS matrix reduces the effect of antibiotics by neutralizing antimicrobial agents or limiting diffusion using extracellular polysaccharides. EPS facilitates intercellular communication, protects cells from chemical damage, provides oxygen diffusion, releases extracellular enzymes for nutrition and, in turn, stimulates the spread of bacteria within the biofilm (Costa et al., 2018; Galdiero et al., 2019). Bacterial biofilms are highly structured, functional, specific and coordinated. Various substances in biofilms coordinate to complete several life activities, such as bacterial biofilms’ morphological diversity, adhesion and protective barrier function. Taking *P. aeruginosa* as an example, **Table 1** shows the main components and basic functions of biofilms.

**Table 1:** Main components and basic functions of *P. aeruginosa* biofilm.

Components	Percentage(%)	Functions	Authors
Exopolysaccharides	1-2	Maintaining the structure and stability of biofilm matrix	Rather et al., 2021
Proteins (including enzymes)	<1-2	Maintaining the stability of biofilm matrix and surface colonization; Maintaining the structural integrity of biofilm	Fong and Yildiz, 2015
Extracellular DNA	<1-2	Promoting biofilm formation; Protecting the integrity of bacterial biofilms; Maintaining structural stability; Protecting the host immune system	Okshevsky and Meyer (2015)
Water	Up to 97	Keeping the biofilm hydrated to prevent it from drying out	Flemming and Wingender (2010)

Bacterial biofilms are communities of microorganisms derived from single or multiple bacterial strains. Compared with single-species biofilms, multi-species biofilms showed the following new characteristics through interspecies interactions: increased biofilm mass, increased community cell count, enhanced metabolic activity of community members, increased antimicrobial tolerance, and changes in spatial organization and structure (Sadiq et al., 2021). Multispecies biofilms exhibit new characteristics different from their floating state, resulting from competition or cooperation. The interactions among bacteria of multiple species are synergy, mutual benefit, cooperation, utilization, antagonism, and competition (Liu et al., 2016). Among them, synergistic interactions play an important role in regulating bacterial microbial activity and constructing complex spatial structures of multispecies biofilms. The cooperation and competition between bacterial cells promote the formation of multispecies biofilms. Multispecies biofilms are found in many natural environments, such as the oral cavity, implantable medical devices, and mammalian intestines. The physical interactions, co-adhesion, and metabolic cooperation among bacterial cells promote the formation of multispecies biofilms in natural environments. Until now, research has focused on single-species biofilms. But now microbiologists are paying more attention to multispecies biofilms and cell interactions between communities. The early stage of multispecies biofilm formation is the adhesion and aggregation of microbial cells. Different biofilm-forming abilities and strategies of various bacteria and microorganisms lead to the formation of multiple types of biofilm (Yao et al., 2022). Multispecies biofilms are associated with developing various diseases, such as cystic fibrosis, diabetic foot ulcers, chronic wounds, and otitis media (Chan et al., 2017; Loera-Muro et al., 2021). The development and outcome of multispecies biofilm-associated diseases are related to the physiological organization and distribution of biofilms.

**Figure 1:**
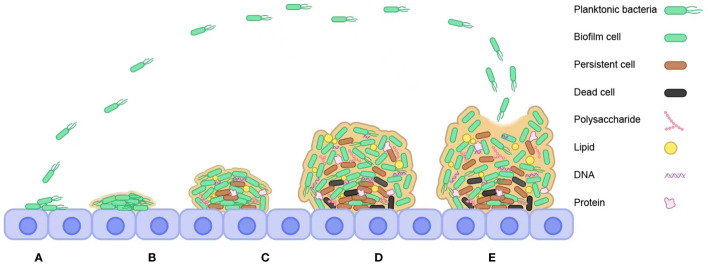
Steps to bacterial biofilm formation. **(A)** Reversible attachment. **(B)** Irreversible attachment. **(C)** Bacterial cells synthesize and secrete EPS. **(D)** Maturation. **(E)** Dispersal.

Due to species diversity, the various stages of bacterial biofilm formation vary but are generally close. The formation of bacterial biofilms is a multi-step process, as shown in **Figure 1**, including molecular attachment to the surface of an object, bacterial adhesion and secretion of extracellular polymeric substances, maturation of the biofilm through the formation of colonies, and bacterial cell escape and dispersion and formation of a new biofilm structure (Kranjec et al., 2021). The process of bacterial biofilm formation is detailed below.

The attachment of bacterial microorganisms to a surface is the first stage of bacterial biofilm formation. The bacterial cells are transported to the object’s surface by convection, Brownian motion, or sedimentation (Palmer et al., 2007). Chemotaxis is prevalent in bacterial microorganisms and refers to the ability of planktonic bacterial cells in fluids to move along a distribution of material concentrations towards chemically induced substances (i.e., sugars and amino acids) or nutrient sources. It promotes interaction between bacterial cells and the surface of an object, which in turn stimulates the colonization and growth of planktonic bacteria on the surface. When planktonic bacteria reach the surface of an object, the sum of the attractive or repulsive forces between the cell surface and the object’s surface determines the interaction between the two characters. When the repulsive force is greater than the attractive force, bacteria cannot adhere to the surface; conversely, when the attractive force is greater than the repulsive force, bacteria can stick to the surface (Carniello et al., 2018). Planktonic bacteria adhere to the surface of objects by non-specific physical forces such as van der Waals forces, electrostatic forces, and hydrophobic interactions (Carniello et al., 2018), and planktonic microbial cells are transformed into stable cells. Still, the process is reversible, and the adhesion is weak. Reversible bacterial cells can maintain a two-dimensional structure Brownian motion and can separate from the surface of an object by their mobility and shear effects. The substrate required for bacterial biofilm growth refers to all substrates that come into contact with planktonic bacteria. Physico-chemical properties such as the substrate surface’s roughness, hydrophobicity, film modulation, and surface charge can influence the efficiency of planktonic bacterial colonization and biofilm formation.

Hydrophobicity plays a vital role in the attachment of bacterial microorganisms to surfaces. A hydrophobic surface is more conducive to bacterial microbial colonization than a hydrophilic material, probably due to the hydrophobic effect, which reduces the repulsion between the bacterial cell surface and the substrate. Yu et al. (2016) suggest that *Streptococcus mutants*’ attachment can influence the hydrophobicity and roughness of the substrate. Bacterial cell surface hydrophobicity is influenced by multiple factors such as bacterial species, growth rate, and culture medium. Hydrophobicity affects the attachment of bacterial microorganisms to surfaces, and when the surface of a bacteria or object is more hydrophobic, the stronger the adhesion between the two. The structural and physicochemical properties of bacterial cells and object surfaces determine whether bacteria attach to hydrophilic or hydrophobic surfaces. Another physical factor affecting bacterial microorganisms’ attachment surface is the surface charge. Many amino, carboxyl, and phosphate groups in most bacterial cells give them a negative surface charge. A positively charged surface facilitates the attachment of planktonic bacteria, while a negative charge hinders the attachment of bacteria (Tuson and Weibel, 2013). The surface charge of bacterial cells is influenced by bacterial species, age, environmental pH, ionic strength, and culture medium. The regulatory film is an integral part of the bacterial adherent surface. Almost all planktonic bacteria come into contact with the regulatory film as they transit from the medium to the surface. It is formed as a nutrient adsorbed onto the surface of an object, resulting in a change in the physicochemical properties of the material surface, which in turn affects the attachment of bacterial cells.

The second phase is irreversible adhesion, where microbial cell surface components can recognize bacterial adhesion molecules and consolidate bacterial interconnections (Nourbakhsh and Namvar, 2016). This phase is accomplished through bacterial cell surface hydrophobicity, hydrogen bonding, covalent bonding, ionic bonding, and dipole-dipole interactions. As bacterial cell surface adhesion structures, flagella and fimbriae can form bacterial biofilms and contribute to the physical contact of planktonic bacteria with the substrate (Carniello et al., 2018). Flagella can swim in liquid substances and swarm on the surface of wet solid substances. Through these two forms of movement, various species of bacterioplankton direct cell adhesion to surfaces and transfer to favorable environments. Flagella initiate the adhesion of planktonic bacterial cells to surfaces, primarily because of the ability of flagella to overcome the resistance that prevents cell-surface interactions. Fimbriae also contribute to the early adhesion of bacterial cells to surfaces and each other. *P. aeruginosa* generates bacterial cell surface motility through fimbriae in a manner known as twitching motility. As an intercellular signaling mechanism, the Quorum sensing (QS) system also significantly facilitates the formation of bacterial biofilms in single cells (Abraham, 2016). Bacterial cells use means through QS to synthesize and release first messengers, such as chemical signals, to enable communication between bacterial flora. Both Gram-positive and Gram-negative bacilli affect the formation of bacterial biofilms. Gram-positive bacilli utilize oligopeptides, while Gram-negative bacilli utilize acyl homoserine lactones (Muhammad et al., 2020).

Bacterial cell synthesis and secretion of EPS is the third stage of bacterial biofilm formation. EPS substrate is hydrophobic and ion bridging, which can promote bacterial condensation and biofilm adhesion. EPS can affect surface adhesion, bacterial biofilm formation, biofilm internal structure, mutual recognition between cells, signal transduction system, nutrient acquisition, cell maintenance, and genetic information exchange, playing a crucial role in various aspects (Costa et al., 2018). The second messenger, c-di-GMP, can stimulate bacterial adhesion from a reversible to irreversible state, mainly due to its ability to produce the EPS matrix and form bacterial cell surface structures. As an essential component of the EPS matrix, extracellular polysaccharides are necessary for most bacteria to form biofilms and promote their development. EPS is also rich in proteins (i.e., enzymes)and protein structures (i.e., fimbriae). At the same time, eDNA and lipids are also components of the EPS matrix. The former can connect to bacterial cells, while the latter can affect the attachment of *Thiobacillus ferrooxidans* (Flemming et al., 2016).

The maturation of bacterial biofilms is the fourth stage of biofilm development. Bacterial biofilms mature as bacteria replicate and multiply in the EPS matrix, forming small microbial colonies and generating three-dimensional structures. As the EPS matrix accumulates and bacterial colonies form, this leads to altered gene expression, and the induction products of these genes can be used for EPS matrix generation. When the matrix is formed, water channels can be generated, which act by a mechanism similar to the body’s circulatory system to deliver available nutrients to the cell community while rejecting extraneous products.

The process of dispersal of bacterial biofilms is the final stage of biofilm development. After a while, the mature bacterial biofilm may suffer damage, and the biofilm structure evolves. Still, the released bacterial microorganisms can infect other parts of the organism and form new biofilm structures. The mechanisms of dispersal are different due to the specificity of the bacteria. Still, all broadly involve the following three standard processes: detachment of bacterial cells from small colonies, transfer of bacterial cells to other substrates, and attachment of bacterial cells to new substrates (Shen et al., 2018). The process of detachment of bacterial biofilms can be either active or passive behavior. Active behavior refers to seeding dispersion, where bacterial cells in the biofilm undergo their detachment to adapt to environmental changes when the bacterial biofilm is subjected to matrix-degrading enzymes, antimicrobials, and nutrient deprivation. Passive behavior refers to shedding and erosion dispersion mediated by external forces (e.g., shear). Shedding dispersion refers to the abrupt shedding of a large proportion of the bacterial biofilm, and erosion dispersion refers to releasing a portion of bacterial cells in a bacterial biofilm. Low expression levels of c-di-GMP can inhibit the formation of bacterial biofilms and promote biofilm separation (Kaplan, 2010). Thus, inhibition of the c-di-GMP signaling pathway can effectively disperse bacterial biofilms. Changes in environmental factors such as temperature, pH, oxygen, and nutrient content can also affect the dispersion process of bacterial biofilms. For example, low-oxygen environments can facilitate bacterial biofilm dispersion by accelerating the rate of c-di-GMP degradation. Increased glucose levels can reduce the amount of c-di-GMP in the organism and promote flagellar synthesis, which slows down the progress of separation (Lee and Yoon, 2017).

The process of bacterial biofilm formation is influenced by temperature and blood pH changes, nutrient content, quorum sensing, Brownian motion, and surface properties. At the same time, different strains and signal transduction also affect the formation of bacterial biofilm. The structure of mature biofilm is a matrix layer, regulatory layer, connective layer and bacterial biofilm layer from inside to outside.

Microorganisms can form biofilms on the surface of the device, causing the development of infectious diseases in the organism. Medical implants have brought hope to the medical revolution and provided new opportunities for human life and health progress. Still, they also increase the risk of infections in the body tissues. Bacterial adhesion is the first step in forming biofilms for device-related infections and is divided into two stages: initial non-specific reversible adhesion and specific irreversible adhesion. *S. aureus* and *S. epidermidis* are the main strains (Pietrocola et al., 2022). These microorganisms can come from the patient’s skin, the healthcare worker’s skin, or the environment. *S. epidermidis* adheres to each other and medical devices by using adhesins. *S. aureus* relies on adhesin ligands (fibronectin, fibrinogen, and collagen) for its adhesion (Darouiche, 2001). The prerequisite for device-related biofilm formation is the coating of the medical device with plasma proteins (Arciola et al., 2018). The type and amount of plasma proteins adhered to the device surface are mainly determined by the surface’s physicochemical properties and the plasma proteins’ characteristics. When *S. aureus* sticks to the surface of a medical device, it can interact through adhesins. Adhesins specifically recognize plasma proteins coated on the surface of the device. *S. aureus* can then proliferate and produce EPS composed of extracellular polysaccharides, proteins, and eDNA. The final stage is when the *S. aureus* community disperses and spreads the infection (Pietrocola et al., 2022). Inhibition of EPS production, enzyme-promoted EPS degradation, and surfactants all contribute to the bacterial dispersion process (Solano et al., 2014). The characteristic change of biofilm dispersion in *S. aureus* is the formation of phenol-soluble modulins (PSM) and extracellular enzymes. PSM plays an essential role in the diffusion stage of biofilm. It disrupts the non-covalent binding forces that strengthen the biofilm matrix, helping to form channels for transporting nutrients to deeper biofilm layers (Peschel and Otto, 2013).

Biofilms are a persistent cause of infection in implanted medical devices. The formation of device-related biofilms is closely related to the interactions between microbial cells. The common microorganisms that can form biofilms on the surface of equipment include *S. aureus*, *S. epidermidis*, *E. coli*, *P. aeruginosa*, *K. pneumoniae*, and *Enterococcus faecalis*. When a medical device is contaminated with these microorganisms, the formation of biofilms depends on several factors. First, the microorganisms must adhere to the exposed surface of the implant long enough to reach the irreversible attachment stage. The adhesion speed of microbial cells on the surface of the device mainly depends on the content and types of bacterial cells in the liquid exposed by the equipment, the liquid’s flow speed, and the physical and chemical properties of the surface. At the same time, the composition of the fluid can change the properties of the device’s surface and affect the rate of cell attachment. When microbial cells irreversibly adhere to the surface of a medical device and produce EPS to form a biofilm, the rate of growth and development is influenced by the flow rate, nutrient content, antimicrobial concentration, ambient temperature, and pH (Donlan, 2001). In the absence of antimicrobial agents, when a medical device is implanted in a host, the surface of the device is immediately surrounded by multiple fluids (e.g., blood, saliva, urine, and other fluids). Mucopolysaccharides, glycoproteins, and metal ions then appear within minutes. These substances can penetrate and adhere to the surface of the device. The bacterial cells can use their surface-specific adhesion molecules to recognize the device surface receptors and thus undergo the adhesion process. Once the microorganism adheres to the device surface, the gene expression level is immediately affected. During the growth and reproduction phase, bacterial cells can synthesize EPS, form microbial communities, and then form biofilms (Huang et al., 2022).

Microorganisms colonize almost all central venous catheters implanted in patients. The most common microorganisms that form catheter biofilms are *S. aureus*, *S. epidermidis*, *P. aeruginosa*, and *K. pneumoniae* (Elliott et al., 1997). These microorganisms can move outward from the skin or inward from the port along the outer surface of the central venous catheter. Colonization of the catheter surface by microorganisms can occur within 24 hours. Biofilms can commonly form on the surface of central venous catheters, but the location and extent of bacterial biofilm formation are influenced by the time of catheter implantation. When the catheter is implanted <10 days, more biofilms can form on the external surface of the catheter. When implanted >30 days, biofilms are more likely to form in the catheter lumen (Raad et al., 1993). Common contaminating microorganisms on urinary catheters are *S. epidermidis*, *E. faecalis*, *E. coli*, *P. aeruginosa*, and *K. pneumoniae*. When the catheter is implanted in the body, these microorganisms tend to form biofilms on the internal and external surfaces. The longer the catheter is left in place, the greater the ability of these microorganisms to form biofilms and the greater the likelihood of causing urinary tract infections.

Microorganisms can form biofilms on non-device (tissue) surfaces. There are several stages of dental plaque biofilm formation. First, the tooth’s surface is covered with an organic “membrane” composed of immunoglobulins, carbohydrates, and glycoproteins. These substances can adhere to the surface of hydroxyapatite through electrostatic interactions. This interaction is generated between Ca2+, phosphate, and molecular groups with opposite charges in saliva. Among other things, carbohydrates comprise intracellularly stored polysaccharides and other intracellularly present polysaccharides. Water-insoluble glucans and fructans promote the attachment of bacterial cells to the tooth surface (Jakubovics et al., 2021). Bacterial cells can bind to surface organic membranes, leading to interactions between adhesion factors and fimbriae, capsules, and complementary receptors. Subsequently, the bacterial cells adhering to the tooth surface can produce various exopolymers to synthesize biofilm EPS (Mirzaei et al., 2020). Maturation and dispersion of dental plaque biofilm is the final stage. During the initial process of dental plaque biofilm formation, the source of nutrition for microbial cells is through the breakdown of salivary substrates (e.g., mucins and other glycoproteins) (Jakubovics, 2015). *P. aeruginosa* is the leading cause of death in patients with cystic fibrosis combined with *P. aeruginosa* infection due to its high virulence factor, biofilm formation, and resistance to antimicrobials. High levels of c-di-GMP expression generally promote matrix production and biofilm formation. In contrast, low levels of c-di-GMP expression down-regulate matrix production and can lead to a bacterial planktonic lifestyle. Carbon starvation and nitric oxide signaling can affect phosphodiesterase activity in *P. aeruginosa* cells, reducing the intracellular expression of c-di-GMP in the cells (Bjarnsholt et al., 2010). *P. aeruginosa* can produce a small molecule that can induce the dispersion of mature biofilms (Davies and Marques, 2009).

Bacterial biofilms provide an excellent and stable homeostasis environment that prevents host immune cells and antibiotics from entering the bacterial biofilm community while protecting bacterial microorganisms from the effects of blood pH, osmotic pressure, and nutrient deficiency. Thus, by providing a physical barrier for bacteria and microorganisms, bacteria can communicate with each other and co-exist, even in harsh conditions. This communication mechanism, called the quorum sensing system, comprises extracellular chemical signals (known as autoinducers). The QS system can help bacteria and microorganisms sense population density and influence biofilm formation and maturation, antibiotic resistance, bacterial communities, and bacterial-host interactions (Whiteley et al., 2017; Paluch et al., 2020). At the same time, some signaling molecules in bacterial microorganisms, such as c-di-GMP, can affect bacterial behavior, including cell cycle, cell movement, pili synthesis, RNA regulation, stress response, and bacterial virulence (Loera-Muro et al., 2021). Bacterial biofilms have specific immune escape mechanisms, including inhibition of immune cell function, alteration of gene expression, obstruction of immune recognition, and mechanical protection (Campoccia et al., 2019; de Vor et al., 2020). When bacterial organisms accumulate on the surface and form biofilms, the clearance function of immune cells is impaired, a phenomenon known as impaired phagocytosis (Urwin et al., 2020).

To provide additional research on bacterial biofilms, we review bacterial biofilm-associated infections, describe current methods used to detect biofilms and effective strategies for treating bacterial biofilms, and give an outlook on the development and future of bacterial biofilms.

## 2 Biofilm-associated infection

Bacterial biofilms can cause serious infections, such as multidrug-resistant, broad-spectrum drug-resistant, and complete drug-resistant bacteria. Currently, more than 80% of bacterial infections are caused by the formation of bacterial biofilms (Fleming and Rumbaugh, 2017). Bacterial biofilms can cause disease in the body through device-related and non-device-related infections. The following will describe the biofilm infections in terms of both and the relationship of bacterial microorganisms to infectious diseases and adhesive surfaces.

### 2.1 Device-related infections

The use of medical devices improves the quality of life for patients. Still, suppose bacterial biofilms form on the surfaces of medical implants (e.g., dental devices, catheters, heart valves, ventricular shunts, joint prostheses). In that case, they may cause bloodstream and urinary tract infections, posing a severe threat to global health. Medical device-associated infections are closely linked to biofilm formation, and the bacterial biofilm on the surface of most devices is composed of a variety of bacteria. Bacterial organisms first attach to the surface of the medical device or the surrounding tissue of the breakage. Bacterial cells proliferate, develop, and form a bacterial biofilm, which is then encapsulated in the EPS. At the same time, the bacterial cells are released from the bacterial biofilm. They can be transmitted through the bloodstream leading to infection or recurrence of localized lesions elsewhere in the body.

Cardiac implants, including pacemakers, artificial heart valves, cardioverter-defibrillators, and cardiovascular implantable electronic devices, are associated with higher morbidity and mortality due to infections (Habib et al., 2013). Gastrointestinal devices are associated with a wide range of microorganisms. A study using scanning electron microscopy showed defects in percutaneous endoscopic gastrostomy cannulas (PEGs), which can provide suitable sites for the attachment of bacterial microorganisms and biofilm formation (Dautle et al., 2003). The study found that bacterial biofilms were present on the surfaces of all devices included in the examination and that PEGs are a risk factor for colonization of the gastrointestinal tract by drug-resistant bacterial microorganisms and the formation of bacterial biofilms. Orthopedic implant surgery is often a safe and efficient treatment modality that restores hip and knee function and enhances patient well-being. However, there are still postoperative complications, the most common of which is an artificial joint infection, on top of which the formation of a combined bacterial biofilm may lead to osteomyelitis.

Neurosurgical implants include cerebrospinal fluid shunts, extra-ventricular cerebrospinal fluid drains, and neurostimulators. The main microorganisms in cerebrospinal fluid shunts are *S. epidermidis* and *S. aureus* (Fux et al., 2006). The microorganisms capable of forming biofilms on extra-ventricular cerebrospinal fluid drains are mainly *S. aureus*, followed by *Propionibacterium acnes* (Strahm et al., 2018). In neurostimulator-associated infections, common pathogenic microorganisms include *S. aureus*, *P. aeruginosa*, and *P. acnes* (Conen et al., 2017). The incidence of infection with these devices is 3-15%, with a higher incidence of infection with extra-ventricular cerebrospinal fluid drains (Caldara et al., 2022). These infections can be fatal to patients and increase morbidity and mortality. Permanent or temporary urinary catheters, nephrostomy tubes, penile implants, and ureteral stents are the most commonly used devices in the genitourinary system. Bacterial biofilms can be found on the surfaces of all these devices. In most clinical situations, infections from implanted stents occur, with fever and urinary tract infections being the most common complications, and even bacteremia and death may occur (Kehinde et al., 2002). Endovascular devices commonly used in clinical practice include intravenous infusions, hemodialysis, haemodilution, and parenteral nutrition. The risk of infection is increased by irregular disinfection, environmental contamination, and incomplete biofilm removal from the surface of implanted devices. To reduce the risk of infection, we should improve disinfection protocols and procedures in the future and detect the propensity for contamination of all types of implanted devices. Breast implants can be used for breast reconstruction and cosmetic surgery, helping improve patients’ quality of life. However, breast implants are susceptible to complications such as infection, hematoma, contracture of the envelope, and scar formation. Contracture can lead to the removal or revision of the implant, causing discomfort and swelling of the breast and affecting its appearance. The researchers detected bacteria in 85% of the breast implants that developed periosteal contracture. They examined them using scanning electron microscopy and found bacterial biofilms in over half of the implants (Pajkos et al., 2003). *P. acnes*, *Streptococcus* spp., *Lactobacillus* spp., *Bacillus* spp., and *Mycobacterium* spp. can survive in the environment around breast implants and form a bacterial biofilm.

Bacteria in the mouth are closely linked to diabetes, heart disease, lung inflammation, and systemic diseases. Prevention and treatment Kalamaraof oral diseases can help prevent these diseases. Oral implants can be used to restore the normal function of the mouth. When bacterial microorganisms colonize the surface of these materials, a solid bacterial biofilm usually forms, leading to inflammation around the implant, which may damage healthy gums. The microorganisms can degrade the composite resin, and the bacteria can invade the implant and tooth interface, leading to severe consequences. Clinicians can apply prophylactic antibiotic treatment to avoid this outcome. At the same time, proper treatment planning, proper implant placement, attention to changes in condition, medication history, and monitoring of underlying health conditions (e.g., diabetes, hypertension, obesity, osteoporosis) are essential to prevent implant failure. Bacterial keratitis is a cornea infection characterized by forming a bacterial biofilm on the eye’s surface. If patients do not receive timely and effective treatment, it will likely lead to vision loss or even loss of vision. Bacterial keratitis is associated with various risk factors, such as corneal trauma, contact lens wear, surgical treatment of the eye, and immunocompromised systemic disease (Ng et al., 2015). Corneal trauma is a significant risk factor for bacterial keratitis in developing countries. Contact lenses are the primary source of bacterial keratitis infection in more developed countries. It can alter the corneal epithelium and carry bacterial organisms to the eye’s surface. Bacterial keratitis is associated with the formation of a variety of bacterial biofilms. The lens materials (e.g., water retention, hydrophobicity) and contact lenses’ physical and chemical properties can influence the colonization of bacterial biofilms. We should follow up with a vigorous search for novel materials to prevent implantable device-related biofilm infections. The most common bacterial organisms on the surface of medical devices are described in detail in **Supplementary Table 1**. In conclusion, medical device implant-related biofilm infections increase morbidity and mortality in patients, especially hospitalized patients, and pose a severe threat to their quality of life. We should pay more attention to the composition of different bacterial microorganisms on the surface of specific medical devices, continue to study biofilms *in vivo*, and systematically describe the interrelationship between bacteria on device surfaces and the surrounding environment of implants.

### 2.2 Non-device-related infections

Non-device-related infections also have a significant impact on health problems. Dental plaque (Rabe et al., 2022), urinary tract infections (Singh et al., 2022), cystic fibrosis (Vandeplassche et al., 2020), otitis media (Bair et al., 2020), infective endocarditis (Han and Poma, 2022), tonsillitis (Klagisa et al., 2022), periodontitis (Prado et al., 2022), necrotizing fasciitis (Grier et al., 2021), osteomyelitis (Huang et al., 2022), infective kidney stones (Fayez Hassan et al., 2021), chronic inflammatory diseases (Wu et al., 2015), bacterial vaginitis (Arroyo-Moreno et al., 2022), and bladder infections (Mirzaei et al., 2020) are all examples of non-device-related bacterial biofilm infections.

The human oral environment, with its favorable temperature and humidity and rich in micro-nutrients, can provide adequate conditions for bacterial growth, survival, and the formation and maturation of dental plaque biofilms. The most common microbial cell grouping in the oral cavity is the bacterial cell, followed by various fungi, viruses, and protozoa. Oral microorganisms co-exist with each other, maintain mutually beneficial relationships with their hosts, and generally do not cause disease. If this community changes, the symbiotic and mutually beneficial balance will be disrupted, leading to various conditions, such as dental caries. Bacterial microorganisms in the mouth can enter the circulation, affect the heart’s function, and bind fatty plaque in the coronary arteries. Dental biofilms can cause periodontitis and gingivitis and even lead to tooth decay. Chronic bacterial infections cause almost all periodontal diseases. Periodontal infections increase the incidence of inflammation, promote the formation of plaque, and lead to edema in the coronary arteries. During the maturation of bacterial biofilms, anaerobes are the main colonizing bacteria in the human mouth. Several studies have shown that dental biofilms can cause not only the development of periodontitis but also various systemic diseases such as diabetes mellitus, infective endocarditis, and rheumatoid arthritis (Larsen and Fiehn, 2017; Marsh and Zaura, 2017). The main bacteria that play a role include *Porphyromonas gingivalis*, *Bacteroides forsythus*, and *Aggregatibacter actinomycetemcomitans*.

*Lactobacillus* strains can be associated with health problems by producing lactic acid to maintain a low pH environment in the vagina, thereby protecting the female genitourinary tract from bacterial microorganisms not of its origin. *Lactobacillus* spp. can play a role in maintaining the homeostasis of the vaginal environment while preventing bacterial microbes from colonizing and infecting it. Women are more prone to urinary tract infections than men because of the proximity of the female urethra to the anus, vagina, and rectum. Disturbances in the body’s vaginal bacterial microbiota also increase women’s risk of urinary tract infections. Estrogen plays a role in maintaining a low pH environment in the vagina, and post-menopausal women are more likely to develop urinary tract infections than younger women due to a lack of estrogen. The most common pathogenic bacteria for urinary tract infections in adult women is *E. coli*. Uropathogenic *E. coli* (UPEC) strains may evade the body’s immune response by stimulating a pro-inflammatory reaction or obscuring the immunogenic bacterial component. Bacterial biofilms may play a key role in maintaining the sustainability of UPEC strains in the bladder and vagina. The second most common pathogen of urinary tract infections is *Proteus* spp. When they first adhere to surfaces, *Proteus mirabilis* can proliferate, develop and form biofilms, enhancing the antimicrobial properties of bacterial microbes and protecting the bacteria from the body’s immune function. Bacterial biofilms are an essential factor in the persistence and existence of bacterial infections, and their diversity can influence the viability and survival of bacterial microorganisms (Delcaru et al., 2016). In addition, bacterial biofilms play an important role in renal pathology and can affect renal stones and the dialysis system. Pathogenic bacteria invade renal tissue and can cause chronic pyelonephritis and bacterial prostatitis. Most of the *E. coli* strains isolated from bacterial prostatitis exhibit the potential to form bacterial biofilms.

Bacterial vaginitis is the most common vaginal infection worldwide and severely impacts the quality of life of women who suffer from it. It is characterized by increased vaginal discharge, usually accompanied by a specific irritating odor. Various bacterial biofilms are present on the surface of the vaginal epithelium. *Gardnerella vaginalis* is associated with bacterial vaginitis infections and has been shown in several studies to produce bacterial biofilms on vaginal tissue and enhance the expression of various virulence factors (Castro et al., 2015; Gaspar et al., 2021).

Cystic fibrosis responds poorly to medications and is one of the chronic inflammatory diseases. Several studies have shown that most people with cystic fibrosis are susceptible to *P. aeruginosa* infections (Harrison, 2007; Filkins and O’Toole, 2015). *P. aeruginosa* infections in combination with cystic fibrosis are challenging to cure, mainly due to the formation of bacterial biofilms in the lungs of patients with cystic fibrosis. *P. aeruginosa* can colonize, develop and form bacterial biofilms in the lungs of people with cystic fibrosis due to the excessive release of mucus in the airways, providing an environment with low oxygen levels. Chronic *P. aeruginosa* infection can lead to complications such as epithelial tissue damage, mucus obstruction of the airways, respiratory dysfunction, and even accelerated death.

Infective endocarditis is a refractory disease that severely threatens human life and health. Although medical technology has been refined, the mortality rate of patients with co-infected endocarditis during hospitalization is still higher than 20% (Beynon et al., 2006). The ability to generate bacterial biofilms is critical in the virulence of bacterial microbes associated with infective endocarditis. Studies (Elgharably et al., 2016; Polewczyk et al., 2017) have confirmed that many bacterial microorganisms can form infectious neoplasms on the surface of the heart, which can lead to the development of infectious endocarditis. These neoplasms are essentially large bacterial biofilms. Using immunofluorescence staining techniques and electron microscopy, Bosio et al. (2012) found *Mycobacterium fortuitum* biofilms on infected artificial biological heart valves. Bacterial microorganisms shed from biofilm structures and enter the blood circulation, and the body will develop bacteremia and sepsis manifestations. Of these, bacteremia is controlled by the collective immune defense system and antimicrobials. Although bacteremia can be effectively prevented or even eliminated with aggressive treatment, the deeper biofilm and neoplasm structures can provide a “hideout” for the ever-present bacterial biofilm cells. When bacterial cells in biofilm structures are repeatedly shed, it is easy to develop septic embolism in the distal limb (Mirzaei et al., 2020).

Otitis media is a complex inflammatory disease, and its complications are a major cause of hearing loss. Approximately 75% of infants under three years suffer from middle ear-associated infections. Bacteria capable of forming biofilm in the middle ear are the leading cause of chronic bacterial infections. Using molecular methods, researchers have demonstrated that the DNA of *Haemophilus influenzae*, *Moraxella catarrhalis*, and *Streptococcus pneumoniae* can be detected in up to 80% of infants with otitis media (Otsuka et al., 2013).

The formation of bacterial biofilms can lead to severe chronic inflammatory and autoimmune diseases in the body. L-type pathogens and chronic biofilm infections can cause the development of inflammatory diseases. The vitamin D receptor is an alkaline substance that regulates the activity of immune cells and is the primary defense against bacterial microbial infections. Specific L-type and biofilm-forming pathogens can produce a substance that adheres to and kills the vitamin D receptor. Thus, as chronic bacterial biofilms and L-type pathogens accumulate in the body, most bacterial microorganisms can produce substances that inactivate the vitamin D receptor and severely compromise the body’s immune defenses. **Supplementary Table 2** describes non-device-related biofilm infections.

### 2.3 Bacterial biofilm cause tissue related and device associated infections

*S. aureus* and *S. epidermidis* can form biofilms on the surfaces of central venous catheters, heart valves, suture devices, and prostheses, in turn, can cause nosocomial infections, endocarditis, mucus cysts, and otitis media (Qu et al., 2010; Arciola et al., 2012). *P. aeruginosa* forms biofilms on the surface of contact lenses, central venous catheter, middle ear, and prosthesis, which can cause nosocomial infection, cystic fibrosis, and otitis media (Wiley et al., 2012; Huse et al., 2013). Bacterial biofilms formed by *S. aureus*, *E. coli* and *Streptococcus agalactiae* can cause mastitis (Loera-Muro et al., 2021). *E. coli* can form biofilms on medical devices such as catheters (Zhang et al., 2019), ultrasonic instruments (Khatoon et al., 2018), and contact lenses (El-Ganiny et al., 2017). The relationship between bacterial microbes adhering to surfaces to form bacterial biofilms and thus causing disease is detailed in **Supplementary Table 3**. The formation of bacterial biofilms will increase mortality in hospitalized patients (Batoni et al., 2016; Evans and Bolz, 2019). Multiple biofilm infections may exhibit different antibiotic sensitivities. Although the specific details of bacterial interactions in numerous microorganisms are not yet precise, there are metabolic links and spatial organization among bacteria, which may result in quorum sensing and resistance gene transfer to produce more antibiotic-resistant biofilms (Røder et al., 2020; Sadiq et al., 2021).

The three-dimensional structure of bacterial biofilm can act as a natural barrier to antibiotics and reduce the sensitivity of biofilm to antibiotics. Bacterial biofilm resistance to antibiotics is known to be 10–1000 times higher than planktonic bacteria (Ning et al., 2014), which may be related to the different antibacterial mechanisms between them. Biofilm communities produce antibacterial target mutations, efflux pumps, higher transverse transfer frequency, reduced cell permeability, and drug-neutralizing proteins (Kumar et al., 2013; Lata et al., 2015; Sharma et al., 2015; Sharma et al., 2016). In addition, there is a particular type of bacterial cell phenotype in biofilms – persistent cells, which can survive under the action of a powerful immune defense system and powerful antibiotics. These cells have metabolic inertia, slow growth and replication, and can regulate virus-antitoxic systems and enhance antioxidant and DNA repair systems. They show an inability to respond to antibiotics (Rather et al., 2021). Anoxia, nutrient deficiencies, antibiotic modification enzymes, and oxidative stress can also lead to antibiotic resistance in bacterial biofilms (Hall and Mah, 2017). Due to the phenotypic diversity of bacterial biofilms, the content of antimicrobials entering the biofilms will be reduced, and the microenvironment within the biofilms will be changed, causing the biofilms’ resistance mechanism to antibiotics (Sharma et al., 2019), making it difficult to eradicate. In addition, genetic mutations can also lead to antimicrobial resistance in bacterial biofilms. As a result, the effect of antibiotics commonly used in clinical practice on bacterial biofilms is insignificant.

In recent years, due to the increasing incidence of iatrogenic bacterial biofilm-associated infections, long-term infections are difficult to cure, which will pose a new challenge to preventing and controlling infectious diseases caused by biofilms. In addition, bacterial biofilms can avoid the scavenging action of conventional antibiotics and the killing effect of the body’s immune system and become a possible source of infection. Currently, methods for detecting, inhibiting and treating biofilm-associated infections are inadequate, and managing bacterial biofilm-associated infections remains a significant challenge. In addition, due to the specific environment within the biofilm, we cannot treat biofilm-associated infections with traditional antibiotics, and researchers must propose new antimicrobial or anti-biofilm-associated treatment strategies.

## 3 Methods for detecting bacterial biofilms

### 3.1 Nuclear medical imaging technology

Nuclear medicine imaging remains the standard method for detecting infectious diseases (Salmanoglu et al., 2018). As radionuclides, technetium-99m, indium-111, and iodine-125 have been shown to be useful for radio-labelling compounds. Still, some drawbacks exist, such as low expression of target receptors on the bacteria studied, non-specific adsorption, and complex radiochemical synthesis (Eggleston and Panizzi, 2014). A recent study found that as a metabolic product of bacteria, the maltodextrin transport system can also radially label compounds and trace them to detect bacterial biofilm infection (Auletta et al., 2019). Nuclear medicine imaging technology also has certain disadvantages, such as the need for specialized equipment and instruments, operator training, and patient exposure to radiation (Cruz et al., 2021).To further improve the sensitivity of nuclear medicine imaging, relevant researchers have developed an MH18F nuclear imaging agent, which can participate in the metabolism of bacterial carbohydrates and be internalized by maltodextrin transporter specific to bacteria (Auletta et al., 2019), contributing to the early detection of bacterial biofilms.

### 3.2 Ultrasonic technology

Ultrasonic technology can monitor dirt on instrument surfaces in real-time and has been shown to monitor the formation and growth of some bacterial microbial colonies (Kujundzic et al., 2007). Combined with other methods, it can enhance the detection strategy of bacterial biofilms (Vaidya et al., 2014). Ultrasound echo enhancers and microbubble contrast agents have opened up new avenues in diagnostic ultrasound medicine (Calliada et al., 1998). Ultrasonic medical imaging technology has matured with the application of contrast media, which contributes to the accuracy of medical diagnosis. The application of targeted ultrasonic contrast media is conducive to determining the difference between healthy and infected tissues (Unnikrishnan and Klibanov, 2012). The acoustic impedance of bacterial biofilms is similar to that of human tissue, making detection and *in vivo* targeting of bacterial biofilm substrates difficult. The combination of ultrasound and targeted ultrasound contrast agents can aid in the early detection and identification of bacterial biofilms and can help to improve therapeutic efficacy. Ligand-targeted ultrasound contrast agents can be a non-invasive imaging method for detecting early and late-stage bacterial biofilms. These agents can target, image, and detect the formation of the *S. aureus* biofilm matrix *in vitro* (Anastasiadis et al., 2014).

### 3.3 Crystal violet staining method

Crystal violet (CV) is the most commonly used staining method for the quantitative determination of microtiters of biofilms grown *in vitro* in polystyrene pore plates (Hassan et al., 2011). After CV staining, the structure of the biofilm can be observed directly by scanning electron microscopy. However, this staining method also has certain limitations, as it can reduce the number of bacterial biofilms after multiple washing (Hou et al., 2022), and the incubation time is longer. It is unsuitable for the rapid detection of biofilms. Castro et al. (2022) found that CV staining was highly effective for single-species biofilm detection. Still, in bacterial vaginosis, there might be bias in evaluating the formation of multiple bacterial biofilms.

### 3.4 Other detection methods

Bioluminescence analysis, tissue culture plate method, and percentage transfer method are also used to detect bacterial biofilms. Certain imaging techniques, such as infrared spectroscopy, reflection spectroscopy, optical fluorescence imaging, confocal laser scanning microscopy, target fluorescence imaging, and fluorescence *in situ* hybridization of peptide nucleic acid, can also be used to detect the formation of biofilms and provide spatial information on the distribution of strains and biofilms (Roy et al., 2018; Cruz et al., 2021). However, these imaging techniques depend on clear samples and are unsuitable for *in situ* bacterial biofilm detection. Raman and surface-enhanced spectroscopy offer high sensitivity and are non-invasive molecular detection techniques (Xu Y. et al., 2020). Laser capture micro-cutting technology has a high resolution, which can help researchers quickly separate or sample the required cells from solid tissue by laser beam, which is helpful for the detection of living biofilms. Ultra-wide-spectrum imaging is a labeling-free detection method showing bacterial biofilms in natural and wound environments.

One of the components of bacterial biofilm is protein, which is related to the types of pathogens and the stages of biofilm development and virulence. Several different proteins are listed below for their role in forming and maintaining biofilm structures. *S. epidermidis* expresses a variety of cell wall-anchored surface proteins that promote the formation of biofilms on the surface of medical devices, aid in binding to the EPS, and are a significant determinant of the virulence of *S. epidermidis*. For example, SdrG/Fbe protein can promote adhesion to the surface of conditioned biomaterials. SdrF protein enhances the adhesion between bacteria and fixed collagen. Embp protein can promote bacterial adherence to fixed fibronectin. Sesl protein may promote adhesion between bacteria and non-living surfaces. Sbp protein can promote the formation of amyloid protein and maintain biofilm integrity (Foster, 2020). McCourt et al. (2014) found that surface-bound fibronectin FnBPA and FnBPB could mediate the formation of methicillin-resistant *S. aureus* (MRSA) biofilm, increase bacterial aggregation, promote initial microbial adhesion on the surface, and facilitate biofilm accumulation. Manfiolli et al. (2018) showed that MAP kinases (MpkA, MpkC, and SakA) and phosphatases could regulate the *Aspergillus fumigatus* cell wall composition and affect cell adhesion and EPS production, as well as have essential effects on signaling pathways during biofilm formation. Liang et al. (2011) identified a novel cell surface protein, BapA1, from *Streptococcus parasanguinis* FW213 and found that it can affect biofilm formation. The BapA1 protein contains multiple putative fimbriae isopeptide junction structural domains that promote the aggregation of bacterial fimbriae in Gram-positive bacteria and is a novel streptococcal adhesin. When the BapA1 protein is deficient, it can inhibit the auto-aggregation of bacterial cells. Ye et al. (2018) found that outer membrane protein W (OmpW) contributed to the survival of *Cronobacter sakazakii* cells in a planktonic mode under the stress of NaCl and that the ability of cells to survive and form biofilms increased with increasing OmpW concentration.

Quantitative proteomics technology based on iTRAQ is helpful for the detection of bacterial biofilms and the search for promising targets for biofilm elimination (Sauer, 2003). The meta-proteomic analysis is an important technique for showing the interactions and functional roles of individual members of bacterial microbial communities. The areas of proteomics include protein function, expression level, post-translational modification, localization, stability, and adequate genome sequencing. Proteomic patterns are responses to the physiological state of cells and can elucidate bacterial biofilm phenotypes (Khemiri et al., 2016). Through the proteomic analysis of *Burkholderia pseudomallei* in the floating state and biofilm state, Khan et al. (2019) found that the change of proteome contributed to the survival of the biofilm by increasing the abundance of pressure proteins and reducing the presence of metabolic proteins. Ali Mohammed et al. (2021) used proteomic analysis to determine the proteome of *Fusobacterium nucleatum* and *P. gingivalis* expressed in the planktonic or biofilm state. The results showed that *P. gingivalis* produces fewer proteins due to the presence of *F. nucleatum* in the mouth. Resolution and dynamic range limit the initial development of proteomics techniques based on electrophoresis. The addition of transcriptomic analysis has led to the rapid development of proteomics because of its ability to detect solid structures capable of detecting functional cells. Charlebois et al. (2016) confirmed that by RNA transcriptional sequencing technology, 25.7% of genes were different between *Clostridium perfringens* biofilms and planktonic cells. About 12.9% of genes in biofilm cells were down-regulated, and about 12.8% were up-regulated. After leptospira forms a mature biofilm, some fundamental biological processes, such as DNA replication and cell division, are down-regulated. Transcriptome-based sequencing techniques can focus on transcriptional changes associated with leptospirosis biofilm formation and maturation (Iraola et al., 2016). *Fusobacterium nucleatum* stain ATCC 25586 at the planktonic cell and biofilm stages by RNA sequencing. It was confirmed that 110 genes of the *F. nucleatum* biofilm state differed from those of planktonic cells. The 85 down-regulated genes in the biofilm state are mainly related to cell proliferation, division, and oxidative stress. The 25 up-regulated genes are primarily associated with amino acid and carbohydrate metabolism (Zhao et al., 2022).

Metabolites are generated in the presence of metabolic enzymes and are the end products of gene expression processes. Many of the life activities in microbial cells (e.g., energy release, cellular signal release, and intercellular communication) are regulated by metabolites. Metabolites can reflect the microenvironment in which a bacterial cell is located and are also closely related to the nutritional status of the cell, the effects of drugs, contaminants, and other external factors. Several metabolites are listed below for their functions in biofilms formed by bacterial pathogens. Glucose, as the primary carbon source and metabolite, can upregulate the expression of extracellular polysaccharide-related gene pslA, which is conducive to promoting the formation of *P. aeruginosa* biofilms and changing metabolic pathways. Glucose could lead to decreased expression of 18 metabolites (including inositol, glutamine, 4-acetyl butyrate, myristic acid, and β-alanine) and increased expression of 7 metabolites (including fructose, 3-hydroxy propionic acid, and glucose-6-phosphate) in *P. aeruginosa* biofilms (She et al., 2019). Guanosine 59-diphosphate 39-diphosphate is also a metabolite that regulates the expression of a large number of genes and plays a vital role in the formation of biofilms of *E. coli*, *E. faecalis*, and *S. mutans* (de la Fuente-Núñez et al., 2014). Short-chain fatty acids, including butyrate, are also strongly associated with the pathogenesis of the periodontal disease. In the initial stage of biofilm formation, butyrate can promote early *Actinomyces oris*-dependent colonization and stimulate biofilm formation (Barbour et al., 2022). *Bacillus subtilis* can produce a variety of specific metabolites. Most of these metabolites (e.g., bacteriolysin, subtilisin A, surfactin, spore-killing factors) are associated with antimicrobial properties (Kalamara and Stanley-Wall, 2021). Schoenborn et al. (2021) investigated the role of nine specific metabolites in biofilm formation. They found that most of them (surfactin A, ComX, subtilin A, spore delaying protein, spore killing factor) could promote biofilm formation.

Metabolomics analysis techniques can reveal the processes of bacterial cell metabolism and are the study of metabolites (e.g., carbohydrates, amino acids, and lipids), intermediate metabolites, and other signaling molecules. The technique emphasizes discrete altered metabolic pathways and highlights biological small molecule metabolites. The Metabolite group is a complete assemblage of all metabolites in the tissues, organs, and compartments of biological cells that are extracted from cells or expressed as bodily fluids (Zhang and Powers, 2012). Metabolomics techniques focus on the broad or total analysis of cellular metabolites through high-throughput detection methods rather than localized and targeted analysis of specific numbers of individual metabolites (Fiehn, 2002). Many metabolic pathways between planktonic bacterial cells and biofilms were changed. Through instrumental analysis, bioinformatics, stoichiometry, and cell biology, metabolomics analysis can show the status of the overall metabolites, simplify the process of metabolite detection, and cover almost all metabolic changes of major pathways. Metabolomics analysis provides a systematic method for characterizing complex bacterial communities, showing the behavior of bacterial cells in biofilms, and contributing to the understanding of biofilms. In addition, biofilms’ strain type and antimicrobial resistance are phenotypic by their differences from the metabolome. Shen et al. (2020) showed that carnosol could inhibit the formation of *S. aureus* biofilm by metabonomics analysis technique.

The fiber optic biosensor is capable of monitoring the growth quality of bacterial biofilms, quantifying analytes, and displaying biofilm properties. It works by monitoring the environment around the sensing element through changes in the refractive index. The sensor offers the following advantages: lightweight construction, compact size, biocompatibility, low fabrication costs, and real-time monitoring. The spherical resonance sensor is highly sensitive and can detect changes in the surface of bacterial cells, such as planktonic cells attached to the surface of an object, and has great potential for early detection of the presence of bacterial biofilms (Rakhimbekova et al., 2022). SiNW-FET, as a new nanosensor, can be combined with microfluidic technology to realize real-time, rapid, and fully automated detection of bacterial biofilms. It can reveal the biological and metabolic processes occurring in bacterial biofilms and has the advantages of high sensitivity, low consumption, non-invasive and traceless (Yeor-Davidi et al., 2020). Dai et al. (2022) reported a PH-responsive branched polymer [poly(MBA-AEPZ)-AEPZ-NA] capable of reducing the dose of antibiotics and overcoming antimicrobial resistance. It can emit intense green light rays in the local bacterial biofilm microenvironment (pH 5.5) to detect biofilm formation in real time. AmyGreen, a water-soluble amino ketone fuel, is a stain that enables the visualization of the amyloid component of the extracellular polymeric substances of bacterial biofilms. It can detect pathological amyloid proteins *in vitro* as a potent fluorescent dye. The application of the AmyGreen stain effectively reduced the risk of false positives when measuring the amyloidogenic fibrils of biofilms. In combination with other stains can be used for confocal fluorescence microscopy (Moshynets et al., 2020). Pandit et al. (2021) have developed a simple sensor of raw, non-functional graphene that is simple to manufacture and can be powerful without the need for precise species identification. It can distinguish and detect different bacterial types according to different growth dynamics, adhesion density, adhesion pattern, and colony formation between bacterial cells, which is helpful for the early detection of bacterial microbial colonization and biofilm formation. A new diagnostic kit is the product of a combination of two reagents, one that relies on a substance that promotes hydrogen peroxide to produce oxygen through catalase and the other a mobile biosensor. The kit can detect *P. aeruginosa* infection in sputum with high sensitivity and specificity within 8 minutes (Clemente et al., 2020). Bacterial biofilms attached to the mucous membranes of the mouth are difficult to visualize with the naked eye. Quantitative light-induced fluorescence (QLF) can detect bacterial infections in the oral cavity and the formation of dental biofilms. Park et al. (2022) demonstrated using QLF to detect and remove pathological biofilms from oral mucosa in elderly patients during hospitalization. The test tube method and Congo red agar technique can help detect biofilm formation by obtaining isolates of bacterial biofilm formation from contact lenses, the conjunctiva of contact lens wearers, and decorative contact lens cases (Raksha et al., 2020). Kouijzer et al. (2021) demonstrated that microvesicles in vancomycin-modified bacteria could detect the formation of *S. aureus* biofilms and could potentially treat *S. aureus* biofilm-associated infections.

Artificial intelligence technologies have also been found to be beneficial for detecting bacterial biofilms due to their powerful computational and learning capabilities. For example, biosensors based on electrochemical impedance spectroscopy can be used to detect *E. coli* biofilms (Xu et al., 2022). Convolutional neural network (CNN) can be used to detect the presence of biofilms and the formation of multiple biofilms through deep learning, with a CNN accuracy of up to 90%. Oh et al. (2022) have developed a material capable of precisely guiding the diagnosis and removal of bacterial biofilms. The material is a magnetic field-directed assembly of nanomaterials into surface topography adaptive robotic superstructures (STARS). It can adapt to complex bacterial surface topography and use automatic motion patterns to target the complex three-dimensional structure of human teeth to detect dental biofilm content with high accuracy while the effect of removing formed biofilm.

Currently, non-invasive techniques used in clinical practice have yet to provide the best method for detecting biofilms. Low practicability, low resolution and low cost-effectiveness limit the development of biofilm detection tools. Therefore, there is an urgent need to develop more accurate and practical detection techniques and diagnostic tools.

## 4 Strategies against bacterial biofilm removal

Bacterial biofilm inhibition is mainly achieved by physical, chemical or biological methods. **Supplementary Figure 1** shows the different therapeutic strategies that inhibit bacterial biofilm formation. Physical methods include ionizing and ultraviolet radiation, damaging instruments and affecting material quality (Galié et al., 2018). Ultrasonic treatment is also one of the physical methods. Its mechanism of action is mainly through chemical and mechanical energy, including pressure, vibration, shear stress, shock waves and agitation. Stable pressure and cavitation can produce multidirectional acoustic microjets, which can damage proximal bacterial microorganisms and their biofilms. Relevant studies have confirmed the effect of plasma technology on therapeutic biofilms (Govaert et al., 2019; Patange et al., 2019). The influence of plasma on bacterial biofilms is mediated by biological activators, such as charged particles, ions, electrons, electric fields, and ultraviolet rays (Lu et al., 2016). Cold plasma can kill bacterial microorganisms and destroy the biofilm matrix. Still, the specific mechanism remains unclear, which may be related to ROS/RNS penetrating bacterial cells, oxidizing and nitrosating lipids and proteins, and then lipid peroxidation, inhibiting enzyme function, and changing DNA structure (Van Impe et al., 2018). Patange et al. (2021) found that atmospheric plasma and acoustic ultrasonic treatment technology can destroy the integrity of bacterial biofilm structure and effectively inactivate *E.coli* and *Listeria innocua* biofilm. They also found that atmospheric plasma was more effective than aeroacoustic ultrasound in treating both early and mature biofilms. When applied in combination, the two technologies have the potential to enhance the inactivation effect. The main mechanisms of atmospheric plasma damage to bacterial biofilms include cell membrane damage, structural changes, and biological and genetic changes. Future studies are needed to understand further the distribution of active particles in atmospheric plasma and acoustic ultrasonic techniques and the detailed mechanism of inactivating bacterial biofilms.

Microneedles (MNs) are an effective and minimally invasive method for the treatment of bacterial biofilms. MNs can not only destroy the tight physical barrier of EPS and directly penetrate the antibacterial agent into the biofilm, but also provide a large specific surface area to promote the diffusion of antibiotics in the biofilm. Yi et al. (2021) combined chitosan and zinc nitrate with MNs structure to produce CS-Zn[II] MNs. This substance has good cytocompatibility, rich acicular design and a large specific surface area, and is conducive to eradicating *S. aureus* and *E.coli* biofilms.

Chemical methods are unstable, cannot play a role in mild conditions, and the price of chemistry is relatively high, prone to producing toxic byproducts. Therefore, it is not usually the preferred method for eradicating bacterial biofilms.

Biological methods have higher inhibition efficiency, which is a relatively new method against biofilm, including the application of bacteriophage, bacteriocin and enzyme treatment of bacterial biofilm. In recent years, emerging anti-biofilm preparations have been developed to limit the adhesion of bacteria on the surface to eliminate the biofilm grown or replace cells from the established biofilm (Batoni et al., 2016). The EPS matrix in biofilms plays a crucial role in evaluating the drug resistance mechanism of biofilms. The ideal anti-biofilm preparation is characterized by its unique structure, antibacterial activity, restriction of EPS accumulation, facilitation of EPS penetration into cells, interference with communication mechanisms between cells, and synergistic action with other antibacterial agents (Wiens et al., 2014).

### 4.1 Antimicrobial peptide

Novel antimicrobial peptides (AMPs), as a kind of cation, can down-regulate biofilm formation genes, prevent bacteria from adhering to the cell-matrix surface, down-regulate QS system signals, and produce a wide range of antibacterial activities against bacterial microorganisms (Yazici et al., 2018). When used alone or in combination with antibiotics, AMPs can effectively inhibit the formation of biofilms or even destroy mature biofilms (Mulani et al., 2019) and can be used to treat biofilm-associated infections. AMPs bind rapidly to cell membranes, reducing bacterial load while circumventing the development of resistance, and it has strong antibacterial, fungal, and viral properties (Pontes et al., 2022). One of the mechanisms of action of AMPs against bacterial biofilms is the down-regulation of gene expression and inhibition of the biological behavior of bacterial cells (e.g., synthesis of DNA, RNA, proteins, and cell walls) (Graf and Wilson, 2019). At the same time, AMPs can interact with signaling molecules, which can control the maturation process of bacterial biofilm and cooperate with antibiotics to resist bacterial microorganisms.

Heinonen et al. (2021) proposed that AMP TAT-RasGAP 317-326 could effectively inhibit the formation and development of biofilms of *P. aeruginosa* and *A. baumannii*, and had little anti-biofilm activity against *S. aureus*. In the human oral environment, AMPs are widely present in the oral mucosa and salivary glands of the epidermal cells and neutrophils (Dale and Fredericks, 2005). AMPs are small in shape and have a positively charged amphiphilic structure, which can enter bacterial microbial cells through transmembrane pores and cause their cracking. AMPs are a potentially effective treatment for bacterial biofilm-associated oral infections due to their extensive antibacterial activity, low drug resistance, and ubiquity in the oral cavity. LL-37 is one of the most widely studied AMPs, which can modulate the immune response, regulate the inflammatory response, accelerate angiogenesis, promote wound healing, and help in oral defense against bacterial biofilms (Wuersching et al., 2021b). Levels of LL-37 are associated with various chronic inflammatory diseases, such as periodontal disease, gingivitis, systemic lupus erythematosus, and psoriasis (Pahar et al., 2020). Lactoferrin peptides are functional AMPs hydrolyzed by pepsin and are an effective anti-biofilm preparation. Wuersching et al. (2021a) demonstrated that LL-37 and human lactoferrin could interfere with the planktonic growth of anaerobic bacteria and the formation of bacterial biofilms, thus reducing the incidence of dental caries and periodontitis. Lactoferrin chimeras have a wide range of antibacterial activities. Ruangcharoen et al. (2017) proved that, compared with minocycline hydrochloride and chlorhexidine digluconate, lactoferrin chimeric could better inhibit the formation of oral multispecies biofilms *in vitro*, and it could reduce the activity of various bacterial cells in the biofilms. Antimicrobials depend on active cells, whereas AMPs are less specific for targeting molecules and can even target metabolically dormant cells in most regions of the mature bacterial biofilm.

At present, treating cystic fibrosis combined with *P. aeruginosa* infection remains difficult. Metal AMP piscidin 1 and piscidin 3 showed activity against *P. aeruginosa* biofilm infection, mainly due to their ability to tolerate an acidic environment and high ionic strength and to cut eDNA in the presence of Cu^2^+. Metal AMP Gaduscidin-1 (Gad-1) is a broad-spectrum AMP that binds Cu^2^+ efficiently in acidic and neutral environments. Holo-Gad-1 can eradicate mature bacterial biofilms and prevent the formation of new biofilms, mainly because eDNA is required for adhesion during the formation of nascent biofilms (Whitchurch et al., 2002). Portelinha and Angeles-Boza (2021) demonstrated that Gad-1 has multiple forms of action and can effectively remove *P. aeruginosa* biofilms in acidic, neutral, and high-salinity environments. When GaD-1 is combined with several commonly used antibiotics (e.g., Kanamycin and ciprofloxacin) to treat bacterial and microbial infections, it can play a synergistic role and improve the survival outcome of patients. This paper lists the acting bacteria and mechanisms of several peptides with antibiofilm activity in **Supplementary Table 4**.

Most AMPs can be combined with antibiotics to enhance the role of antimicrobials in preventing bacterial biofilm formation and killing mature biofilms. Antimicrobial peptides are expected to improve the antibiofilm effect and reduce the dosage of antibacterial agents. However, there are limitations to the application of AMPs. Because AMPs are trapped by anionic biofilms, are easily broken down by enzymes in the biofilms, and may even cause acute hemolytic and toxic reactions.

### 4.2 Nanoparticles

Nanomaterials can reduce the adhesion of bacterial biofilms, promote the delivery of antimicrobial agents, improve the permeability of antibiotics, maintain the stability of antibiotics, and directly produce resistance to biofilms through specific mechanisms without antibiotic treatment. Due to their unique physical and chemical properties, namely biological response, surface charge and small-scale effect, nanomaterials can acquire a variety of antibacterial modes and perform multi-potency interactions with bacterial cells. Nanomaterials are effective carriers of antibacterial agents and inhibit the growth of biofilms through thermal damage, oxidative stress, and physical damage, which is conducive to the treatment of bacterial biofilm infection (Makabenta et al., 2021), and are not prone to drug resistance (Pelgrift and Friedman, 2013). Because of their special structure, nanomaterials can increase antibacterial activity in the following four ways. The surface charge of nanomaterials can enhance the interaction with bacterial microbial cells and lipid molecules, thus extending the exposure time of bacterial cells to antibiotics. Some nanomaterials reduce the generation of bacterial drug resistance through the self-cracking mechanism. Nanomaterials can enhance antimicrobials’ solubility, prolong antimicrobials’ life cycle, maintain the effective release of antimicrobials at the target site, and deliver multiple antimicrobials to the same target site to achieve combined therapy (e.g., photothermal and photodynamic therapy). Some nanovesicles inhibit the pre-degradation or release of drugs and deliver antimicrobials to designated targets *via* membrane fusion mode (He et al., 2022). **Supplementary Figure 2** shows the mechanism of action of nanomaterials for inhibiting bacterial biofilm formation.

It is known that most organic nanoparticles can improve the dispersion performance of bacterial biofilms and have good biocompatibility, which is an important research direction in the field of antibacterial biofilms. Nanomaterials provide a new idea for the treatment of bacterial biofilms in the future, which can effectively improve the therapeutic efficiency of antibiotics and reduce the drug resistance caused by biofilms. The therapeutic effect of nanomaterials in the treatment of bacterial biofilms is mainly affected by their unique physical and chemical properties and the characteristics of biofilms. The combined application of organic nanomaterials with different functions can improve the efficacy of anti-biofilm. New composites composed of organic nanomaterials and other materials with antibacterial biofilm activity can significantly enhance the ability to resist biofilms and are currently the most widely used antibacterial substances.

Nanoparticle-based therapeutic regimens are expected to be effective for removing bacterial biofilms because of their advantages: functional versatility, selectivity, traceability, high loading efficacy, and controlled drug release (Datta et al., 2018; Khan et al., 2018). Unlike conventional antimicrobials, the activated nanoparticles can be designed to work in the presence of only a trigger, which reduces the side effects of being off-target (Schwartz-Duval et al., 2020). Therefore, the active development of nanomaterials for treating bacterial biofilm-associated infections has important research implications (Gao et al., 2020M; Yu et al., 2020). Ostadhossein et al. (2021) proposed a “particle-in-particle” treatment scheme for the first time. In the absence of antibiotics, they can deliver small therapeutic nanoparticles through simple chemical methods, which can target the characteristic pH of EPS, promote the killing of bacteria mediated by bacterial biofilm pH, and then use nanoparticles to kill caries pathogen S. Under the low pH value, the metabolic state of dental biofilm can produce pathogenicity. Targeting this factor can stabilize the original ecological balance and inhibit harmful pathogenic microorganisms. Particle-in-particle therapeutic approaches have been shown to provide excellent drug delivery, especially during oncology treatment (Xu F. et al., 2020; Zhang et al., 2020). Carbon dots (CDots), a new class of carbon-based nanoparticles, have the advantages of a simple fabrication process, adjustable luminescence function, and wealthy off-energy groups (Nguyen et al., 2020), which can provide unique conditions for antibacterial biofilms. One study confirmed that CDots could reduce the amount of caries pathogen S while killing bacterial biofilm EPS. *In vivo*, nanoparticles can effectively kill *S. mutans* and balance the oral environment (Ostadhossein et al., 2021).

Nanomaterials are effective in preventing the formation of bacterial biofilms on the surface of implantable medical devices. Metal nanoparticles are the key types of nanoparticles that have intrinsic anti-biofilm activity. Some metallic nanomaterials interact with the EPS of bacterial biofilms through surface charge interactions, and they can release soluble ions targeting bacterial microorganisms or EPS (Hiebner et al., 2020). Silver is one of the metal nanoparticles with high sterilization capacity. Silver nanoparticles (Ag-NPs) effectively prevented biofilm formation of *S. aureus* and oral mixed flora (*Streptococcus oralis*, *P. gingivalis*, and *Actinomyces naeslundii*) biofilm formation. Ag-NPs with maximum antibacterial activity after three repeated irradiations (Pérez-Tanoira et al., 2022). Habimana et al. (2018) developed a substance (GNP+PK) that treated gold nanoparticles (GNP) with the enzyme protease K (PK). They treated the bacterial biofilm on the surface of medical devices for 24 hours with enzyme protease K, gold particle, and GNP+PK, respectively. The total biomass of *Pseudomonas fluorescens* biofilm decreased by 40%, 77%, and 74%, while the thickness was reduced by 52%, 72%, and 78%, respectively. This study confirmed that GNP+PK particles could kill cells in *P. fluorescens* biofilm, mechanically separate cells in suspension, and damage the structure of bacterial biofilm, showing better anti-biofilm efficacy than PK or GNP. Slomberg et al. (2013) combined NO and silica nanoparticles to examine the effects of these materials on *S. aureus* and *P. aeruginosa* biofilms. In this process, the bacterial biofilm was first placed in a biological reaction incubator for 48 hours and then treated the biofilm with nanoparticles for 24 hours. They found that the morphology and volume of nanoparticles both affected the effectiveness of bacterial biofilm elimination. Chitosan (CS) carries a positive charge on its surface and can adsorb to the surface of bacterial microorganisms. It binds to the surface of bacterial cells carrying a negative charge using electrostatic interaction, and the permeability of bacterial cell membrane changes, leading to apoptosis (Rashki et al., 2021). At the same time, chitosan can penetrate the bacterial cell membrane and interact with nucleic acids to interfere with the DNA/RNA synthesis process, triggering intracellular reactions that lead to cell death. Pan et al. (2022) combined CS with artificial nano enzymes. They found that the conjugate is an efficient, economical, and environmentally friendly antibiofilm preparation, which may eventually become an effective alternative to eradicate bacterial biofilms.

Nanosponges can prevent the formation of biofilms or eliminate biofilms those already formed and exhibit broad-spectrum antimicrobial membrane activity against pathogenic single and double-species biofilms, with no toxicity to mammals. Nabawy et al. (2021) reported the ability of biodegradable polymer-stabilized oil-in-water nanosponges to inhibit methicillin-resistant *S. aureus* and *P. aeruginosa* biofilms. Raj et al. (2022) synthesized chitosan-gum arabic-coated liposomes-alizarin nanocarriers. They found that this material can eliminate the biofilm formed by *S. aureus* and *Candia albicans*, which helps improve drug penetration and release in biofilm cells. However, low stability and water solubility limit the broad application of nanomaterials. At present, a new method has been proposed for the treatment of biofilm-associated infections, namely “nanoscale bacterial debridement”, which can separate bacteria from biofilm in a specific way, effectively kill bacteria, and reduce the biomass of biofilm (Li et al., 2021). However, as a new type of nanomaterial, “nanoscale bacterial debridement” has received little relevant research. Biofilm removal by separating bacteria and biofilm has broad research prospects and needs further investigation. Most nanomaterials have been shown to be effective solutions for preventing bacterial biofilm formation (Baelo et al., 2015; Cheeseman et al., 2020). Since biofilms exhibit a high antibacterial bacterial microbial community embedded in EPS, not all nanoparticles will be able to destroy bacterial biofilms. Although organic nanomaterials play an excellent role in inhibiting the formation of bacterial biofilms, they are less effective against stationary and persistent bacteria (Li et al., 2021). The cytotoxicity and abnormal distribution of complex tissues are common problems in nanomaterials application. Therefore, more in-depth research is needed to focus on the overlooked aspects of bacterial biofilm processing.

### 4.3 Catabolite control protein A inhibitor

Catabolite control protein A (CcpA) is the primary regulator of the trans-activation of carbon catabolite repression. It can induce and stimulate the upregulation of cidA and icaA gene expression. The cidA and icaA genes can play a role in synthesizing polysaccharide intercellular adhesins. They can be involved in encoding cave proteins that are involved in lysing bacterial cells and releasing eDNA (Sadykov et al., 2019). At the same time, CcpA can inhibit small noncoding RNA RsaI transcription and then affect bacterial biofilm formation (Bronesky et al., 2019; Bulock et al., 2022). CcpA of *S. aureus* (SaCcpA) can also affect bacterial biofilm formation, antimicrobial resistance development, and virulence factor expression. Catabolite control protein A inhibitor can inhibit the DNA-binding ability of SaCcpA, which is expected to eliminate the virulence factor of *S. aureus* strains. Silver, as an inhibitor, can target SaCcpA, eliminate alpha-hemolysin expression in bacterial cells, and reduce bacterial virulence (Liao et al., 2017). However, silver ions are toxic, and silver-containing preparations (e.g., silver sulfadiazine) are generally used only as topical treatments. Huang et al. (2020) identified a specific small molecule inhibitor that disrupts the SaCcpA-DNA complex product and reduces the expression of α-hemolysin encoded by the hla gene. The Sak gene is a staphylokinase that can convert plasminogen into plasmin (Tam and Torres, 2019). It can inhibit the formation and development of biofilms and separate mature biofilms by activating plasminogen and splitting fibrin from the host. After treatment with the Sak gene, *C. albicans* and *S. aureus* biofilms integrity and biomass decreased (Liu et al., 2019). Zheng et al. (2022) proposed that CcpA can bind to the promoter region of the Sak gene and inhibit Sak gene expression, thus regulating the formation and development of bacterial biofilms. This method has low cost and minor side effects and is expected to be a drug for treating *S. aureus*-related biofilm infections.

### 4.4 Bacteriophage

Bacteriophage is a virus that can infect and kill bacteria. Compared to common antibiotics, bacteriophage has the advantages of functional specificity, more robust tolerance, higher safety, narrower scope of action, and cost-effectiveness (Principi et al., 2019), and it cannot infect human or animal cells. Bacteriophages can penetrate the structure of bacterial biofilm and eradicate or prevent bacterial biofilm (Łusiak-Szelachowska et al., 2020), which is expected to be an alternative therapy for antibiotics. **Supplementary Figure 3** shows the multiple mechanisms by which bacteriophages counteract bacterial biofilm formation. Haemolysinase is a phage-derived enzyme that can hydrolyze the peptidoglycan of the cell wall, which in turn can disrupt the structure of bacterial biofilms (Gray et al., 2018; Sharma et al., 2018). Bacteriophage infection of host cells mainly involves the following steps: (i) adhesion to the bacterial cell surface using phage receptor binding proteins; (ii) the phage genome enters the cytoplasm; (iii) phage for protein assembly; (iv) release of progeny phage (Tian et al., 2021).

Novel phage Φ 15 produces a polysaccharide depolymerase that hydrolyzes the EPS of a single species of single-species *Pseudomonas putida* and inhibits biofilm formation (Cornelissen et al., 2011). Rai et al. (2020) used CV staining to determine the biofilm content. They found that phage PD1 and PE2 could effectively prevent the formation of *S. aureus* biofilms, while PD1, PE1, and P3 could disperse mature biofilms. Phage Φ29 and phage T4 could inhibit the formation of *S. aureus* biofilms (Sybesma et al., 2016). Phages from the order Caudovirales, Myoviridae family, could reduce the proliferation of *P. aeruginosa* in the floating state. At the same time, this phage could also reduce the metabolic activity of endotracheal cannula-associated biofilm and destroy the already-formed *P. aeruginosa* biofilms (Oliveira et al., 2020). The phage mixture (LPSTLL, LPST94, LPST153) has a wide host range and lytic activity, which can play a role in reducing the biofilm of *Salmonella* spp. (Islam et al., 2019).

Combining bacteriophage and antibiotics can improve the therapeutic effect of bacterial biofilms and achieve reduced phage resistance without increasing the toxicity of antimicrobials. For example, the combination of bacteriophage T4 and tobramycin can significantly reduce *E. coli* tolerance to tobramycin (Hemmati et al., 2021). Combining specific phage and amoxicillin can improve the synergistic effect on *K. pneumoniae* B5055 biofilm (Bedi et al., 2009). Bacteriophages can be used alone or in combination with various bacteriophages in treating bacterial biofilms. Phage mixtures are effective in preventing the formation of bacterial biofilms and removing mature biofilms because they produce fewer types of phage resistance and significantly increase the host spectrum compared to phage alone. However, bacteriophage therapy also has limitations: for example, bacteriophages increase antibiotic resistance, bacteriophage DNA replication and protein synthesis need to interfere with bacteria metabolism, and bacteriophages are related to the growth conditions of bacterial microorganisms (Tagliaferri et al., 2019; Łusiak-Szelachowska et al., 2020). Since bacteriophages are viruses and pathogenic factors, there may be potential hazards. Therefore, when combining phages and antibiotics for treating bacterial biofilms, we should fully consider the possible adverse effects to prevent contraindications to the pairing.

### 4.5 Quorum sensing inhibitors

The communication mechanism between bacteria and microorganisms is called the quorum sensing system, which can control the expression of various virulence genes at different stages of bacterial biofilm formation and development (Dijkshoorn et al., 2007). Inhibition of this system can impair the formation of bacterial biofilm. As a small signal molecule, an autoinducer can mediate the communication between bacteria and microorganisms in the QS system to coordinate the development of bacterial cells. Significant changes in intracellular gene expression levels may occur when bacterial population densities reach concentration thresholds set by autoinducer agents while under the influence of the external environment (Hemmati et al., 2020). Gene expression level can induce or inhibit various virulence factors in bacterial cells and affect biofilm formation. Autoinducer peptides (AIP), Autoinducer-2 (AI-2), and N-acyl-homoserine lactones (AHLs) are three of the most studied QS signaling molecules (Muras et al., 2022). AIP is synthesized by Gram-positive bacteria. It cannot penetrate bacterial cells but binds to specific transmembrane receptors in cell membranes or cells to stimulate signal transduction pathways and promote the transcription of target genes. AHLs are usually produced by Gram-negative bacteria and can spread into bacterial cells to bind to specific receptor proteins that activate corresponding transcription factors and regulate gene expression (Duplantier et al., 2021). Blocking the production of AHLs can reduce the biological activity of AHL synthase and decrease the concentration of AHLs, which in turn interferes with the QS system. The AI-2 molecule is an autoinducer that mediates the signaling process between Gram-negative and Gram-positive bacteria, which is influenced by osmotic pressure and pH. The signaling molecule will be broken down when exposed to low osmotic pressure. AI-2 signaling molecules can affect bacterial biofilm formation, antibiotic resistance, virulence factor secretion, and cell motility Li and Zhao (2020).

Quorum sensing inhibitors (QSI) are produced by prokaryotes or eukaryotes. It can block the quorum sensing system, which may reduce efflux pump expression and destroy bacterial biofilm formation (Hemmati et al., 2020). QSI has been shown to interfere with AI-2 and the Competence Stimulation Peptide system to prevent the formation of oral bacterial biofilms (Muras et al., 2020). Non-steroidal anti-inflammatory drugs (e.g., Meloxicam and aspirin) can be used as potential QS inhibitors, which can affect the QS signaling molecules of *P. aeruginosa* and the formation and maturation of its biofilm (Almeida et al., 2018). Some commonly used antimicrobials (e.g., erythromycin, azithromycin, piperacillin-tazobactam, streptomycin, and ciprofloxacin) have high levels of QSI activity (Skindersoe et al., 2008). Natural products, antibiotics and compounds can affect the function of QSI. Bacteria may be resistant to a single QSI preparation, reducing its effective biological activity. Bacteria may be resistant to a single QSI preparation, reducing its effective biological activity. Therefore, it is recommended to combine the application of QSI and antibiotics to inhibit resistance to QSI without increasing the toxicity of antibiotics (Wang et al., 2016), which can effectively improve the therapeutic effect. When resveratrol was combined with aminoglycoside antibiotics (gentamicin, streptomycin, amikacin) to treat bacterial microorganisms, it significantly reduced the formation of bacterial biofilms compared with various substances alone (Zhou et al., 2018). Therefore, QSI can target the quorum sensing system in bacteria and may be a potential therapeutic solution for bacterial biofilms.

### 4.6 Enzymes involved in biofilm degradation

Enzymes are involved in ESP generation, intercellular communication, maturation and dispersion of biofilms, with high specificity. It can remove specific components from biofilms, helping to inhibit the extracellular matrix and quorum-sensing system of bacterial microorganisms (Ivanova et al., 2015). Enzymes capable of hydrolyzing ESP components have anti-biofilm activity, including oxidase (Nguyen and Burrows, 2014), polysaccharide degrading enzyme (Saggu et al., 2019), and proteolytic enzyme (Wille and Coenye, 2020). Therefore, enzymes are preferred among biological methods for inhibiting biofilms (Tan et al., 2020). **Table 2** shows several enzymes and their mechanisms of action.

**Table 2:** Enzymes with antibacterial biofilm activity.

Enzyme	Mechanism of action	Bacteria	Authors
Trypsin	Hydrolysis of the peptide bond on the carboxyl side of protein arginine and lysine residues	Inhibition of biofilm formation in *P. aeruginosa* and *Erysipelas rubbery* C208	(Gilan and Sivan, 2013; Banar et al., 2016)
Cellulase	Hydrolyses the β-1, 4 glycosidic bonds of hemicellulose and cellulose	Decomposition of EPS in *P. aeruginosa* biofilms	(Ibrahim et al., 2021)
α-amylase	Hydrolysis of α-1,4 glycosidic bonds in glycogen and starch		(Fleming et al., 2017)
Pectinex ultra clear	Contains polygalacturonase, pectin ester, hemicellulase and cellulase activity		(Perwez et al., 2021)
Disintegrin B produced by Aggregatibacter actinomycetem-comitans	Hydrolysis of glycosidic bonds in polysaccharides	Decomposing the mature S*taphylococcus* spp. biofilms	(Donelli et al., 2007)
Alginate lyase	Resistant alginate substances in *P. aeruginosa* biofilm substrates	*P. aeruginosa*	(Franklin et al., 2011)

Soluble enzymes are unstable under different biofilm growth conditions and are not reusable. Enzyme immobilization can achieve enzyme stability, improve utilization, and reduce activity loss (Perwez et al., 2017). The immobilization includes adsorption, crosslinking, embedding, encapsulation and covalent coupling. Perwez et al. (2021) proposed m-combi-Cross linked Enzyme Aggregate (m-combi-CLEA), a novel biofilm inhibition method that can inhibit the biofilms of *E. coli* and *S. aureus*. The bacterial biofilms treated with enzyme mixtures in the form of CLEA showed inhibition rates could reach 75-78%, promising an alternative to antibiotics. The magnetic effect of CLEA helps achieve enzyme recirculation.

### 4.7 Aptamers

Aptamers are single-stranded oligonucleotide molecules or peptides produced *in vitro*. Because of its specific three-dimensional structure can select target molecules (e.g., cells, proteins, antimicrobial agents, small molecules, and metal ions) by specific linkage and high affinity. These properties make the aptamers highly active against bacterial biofilms and antimicrobials. Their antibacterial effects may be generated through the depolarization of cell walls of bacterial microorganisms. Aptamers may be an effective alternative to inhibit the development of biofilms (Shatila et al., 2020). **Supplementary Figure 4** briefly shows the primary mechanism of action of aptamers in inhibiting bacterial biofilm formation. Aptamers are suitable alternative substances in the treatment of biofilms due to their flexible diagnostic and therapeutic properties. Mao et al. (2018) developed an aptamer-targeted graphene oxide (GO) therapeutic strategy against bacterial biofilms, defining the coupling as aptamer-GO. They found it can inhibit 93.5 ± 3.4% of *Salmonella typhimurium* biofilms, showing superior antibacterial biofilm properties and effectively becoming a long-term therapeutic option for treating bacterial biofilms. As a special targeting agent, aptamers are expected to improve the effective concentration of antibiotics and reduce the possibility of missing targets, which can be used to treat bacterial microbial infections. The synergistic action of aptamers and antibiotics may affect more bacterial microbial cells (Ning et al., 2015).

Shatila et al. (2020) demonstrated the inhibitory effect of DNA aptamer of *Salmonella* spp. invasion protein A (Apt177) on bacterial biofilms. They found that Apt177 could alter the three-dimensional structure of biofilms and was effective in reducing bacterial biofilm formation when applied alone or in combination with ampicillin. Enteropathogenic *E. coli* (EPEC) is a biological agent that causes diarrhea by forming a bacterial biofilm. It was found that the nucleic acid aptamer SELEX 10 Colony 5 could reduce the motion diameter of EPEC K1.1 and showed the highest biofilm inhibition effect. This aptamer can reduce the mRNA expression level of bacterial biofilm formation genes (e.g., curli gene, interaction, and motility), hinder EPEC K1.1’s motility, and prevent bacterial biofilm formation (Oroh et al., 2020). Sengupta et al. (2020) reported the effectiveness of aptamer-DNA template silver nanocluster (Ag-NC) for inhibiting *P. aeruginosa* biofilm formation, and Ag-NC as a sensor is expected to be a new way to detect planktonic cells and biofilm formation. Ning et al. (2022) found that simultaneous delivery of penicillin-binding protein 2a-specific DNA aptamer and berberine *via* graphene oxide effectively inhibited the formation of MRSA biofilms, and this complex could potentially be an effective strategy for the treatment of chronic infections caused by MRSA biofilms. *P. gingivalis* can cause the occurrence of periodontitis. DNA-aptamer-nanographene oxide (NGO) achieves real-time, *in situ* detection and removal of *P. gingivalis* biofilm. The DNA-Aptamer-NGO complex serves as an antimicrobial photodynamic therapy and is a promising method that may inhibit the formation of bacterial biofilms (Pourhajibagher et al., 2022). Therefore, aptamers combined with other reagents can improve targeting specificity and inhibit the formation and development of bacterial biofilms, which is considered an ideal measure for antibacterial biofilms.

### 4.8 Peptide nucleic acid

Peptide nucleic acids (PNA) is a synthetic analogue of nucleic acid, composed of nucleic acid and peptide, similar in structure to DNA or RNA, and identical in physical and chemical properties to protein. PNA has a high affinity and specific binding ability, showing great potential in removing drug-resistant bacteria (Lee et al., 2019), and can hinder the formation of bacterial biofilms. Combined with conventional antibiotics, it can improve the antibacterial efficacy of antibiotics and anti-biofilm activity. There are two main reasons why pre-PNA has yet to be widely used. One is that PNA is hydrophobic and not easily dissolved in an aqueous solution. Second, due to the lack of effective carriers to transport PNA to biofilms, bacterial biofilms have low permeability to PNA (Wojciechowska et al., 2020). Meanwhile, components of bacterial cells, such as lipopolysaccharide and peptidoglycan, are also major barriers that restrict PNA entry into biofilms (Kurupati et al., 2007). Various methods have been proposed to increase the penetration of PNAs into biofilm cells: chemical changes in the structure of PNAs can enhance the hydrophilicity of PNA. Some cell-penetrating peptides (e.g., chloroquine, photochemical internalization, cationic lipids) can covalently bind to PNAs. This process helps PNAs form complementary base pairs with bacterial DNA. Filamenting temperature-sensitive mutant Z (FtsZ) remains silent in numerous bacterial cells and is one of the essential structures involved in the bacterial division process, making ftsZ a potential target for developing new antimicrobial agents. Narenji et al. (2020) showed that antisense PNAs targeting the efaA gene could reduce the formation of *Enterococcus* spp. biofilms, that anti-ftsZ materials could inhibit bacterial cell growth by interfering with *E. coli* division, and that PNAs were shown to inhibit the function of ftsZ. AcpP-PNA14-5’L is a PNA peptide based on targeting the acpP gene with a 5’ membrane penetrating peptide and junction. It exhibits efficient antibacterial activity against *H. influenzae* in both planktonic and biofilm states and is not susceptible to drug resistance (Otsuka et al., 2017). Castillo et al. (2018) found that PNA can directly block the transcription of mRNAs encoding acyl carrier proteins. Certain antibiotics (such as polymyxin B) can interfere with cell wall formation and, when used in combination with PNA, are effective in preventing the formation of biofilms from Enterohemorrhagic *E. coli* 0157: H7. The combination of PNA with conventional antimicrobials has the potential to be an effective therapeutic option for the treatment of Gram-negative bacteria. In conclusion, PNA is expected to be an effective way to increase anti-biofilm activity.

### 4.9 Vaccine

Currently, vaccines formulated using biofilm-derived antigens are an effective way to prevent infectious diseases, which can improve the protective efficacy of existing vaccines (Loera-Muro et al., 2021). The outer membrane vesicles (OMV) derived from *Bordetella pertussis* can effectively prevent the colonization of bacterial cells in the lung of mice. Carriquiriborde et al. (2021) used OMV from the planktonic state (OMVplank) and biofilm state (OMVbiof) to create a vaccine. The OMVbiof vaccine was more affinity and immunogenic than the OMVplank vaccine in antibody induction. In addition, the *B. pertussis* biofilm-derived vaccine was found to be more protective and immunoreactive against bacterial strains with defective pertactin antigen expression than the OMVplank vaccine. Zurita et al. (2019) found that the OMVs vaccine induced respiratory CD4 tissue-resident memory cells with long-lasting protection against *B. pertussis*. It caused a durable immune response, making it an excellent alternative to third-generation pertussis vaccines. The treatment of pathogens with biofilm-derived vaccines is still in the research stage and may help treat biofilm infections. More advanced technologies are needed to identify bacterial biofilm-derived antigens in the future.

## 5 Conclusion and prospect

Clinically, most chronic infections are associated with biofilms of bacterial microorganisms, which are resistant to antibiotics and can grow and mature even under poor survival conditions. Biofilm-associated infection is a significant clinical problem. Biofilms can form on the surface of devices and non-devices, increasing patient morbidity and mortality and seriously threatening human life and health. In addition, the increased spread of multidrug-resistant bacteria has made biofilm infections a significant threat to hospitalized patients. Although many studies have been done to elucidate devices and non-devices surface biofilm formation, they are mainly limited to a few surfaces and specific bacterial microorganisms. Therefore, it is critical to focus on bacterial biofilm infections and work to raise awareness of the different microbial populations present on device and non-device surfaces to develop strategies for detecting and treating bacterial biofilms. Early detection of bacterial biofilms can improve treatment effectiveness and reduce medical costs. There are many strategies to resist biofilms, but relatively few to clinical treatment. In the future, we should also focus on more efficient, durable, and environmentally friendly methods and further study the safety and effectiveness of each strategy.

## Author contributions

Conceptualization- YL; collecting data- AZ and JS; writing and editing- AZ; supervision- YL. All authors contributed to the article and approved the submitted version.

## Supplementary material

Supplementary Figure 1 Different therapeutic strategies for inhibiting bacterial biofilm formation.

Supplementary Figure 2 Mechanism of action of nanomaterials against biofilms.

Supplementary Figure 3 Mechanism of action of bacteriophages with anti-biofilm activity.

Supplementary Figure 4 Mechanism of action of aptamers with anti-biofilm activity.

Supplementary Table 1 Most common bacterial organisms on the surface of medical devices.

Supplementary Table 2 Non-device-related biofilm infections.

Supplementary Table 3 Microbial and bacterial biofilm-associated infections and their adhesion surfaces.

Supplementary Table 4 Mechanism of action of antimicrobial peptide.
