# Supplementary figures and images for: Understanding bacterial biofilms: From definition to treatment strategies
Source: Front Cell Infect Microbiol. 2023 Apr 6;13:1137947. doi: 10.3389/fcimb.2023.1137947 (PMC10117668; doi:10.3389/fcimb.2023.1137947)

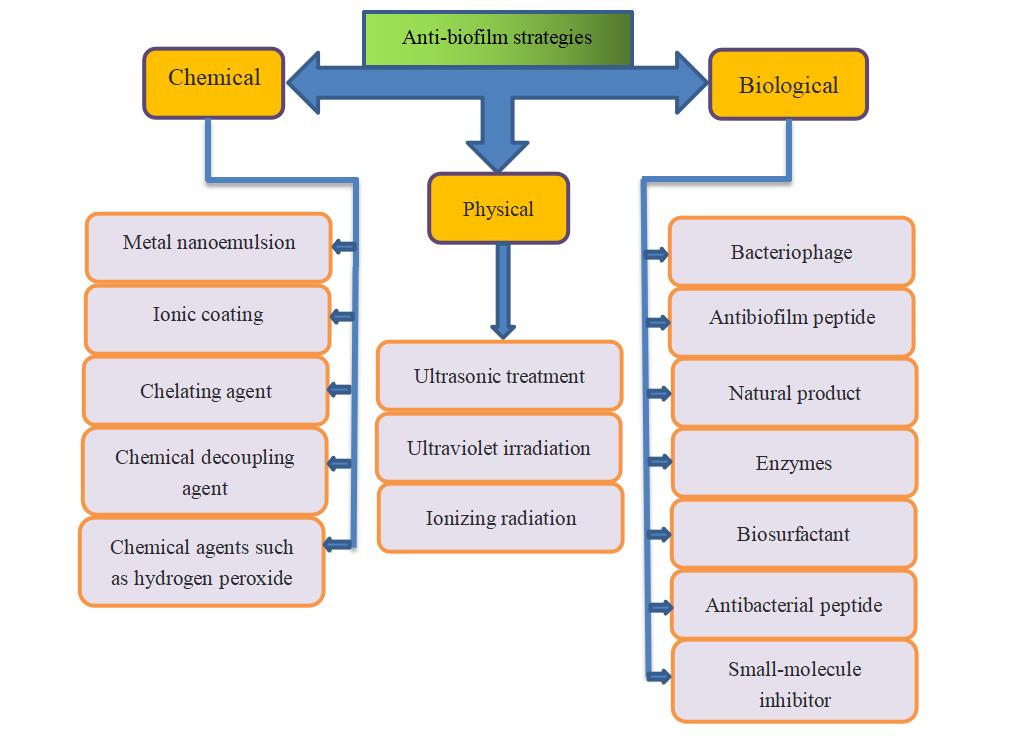

Supplement: Supplementary Figure 1 — Different therapeutic strategies for inhibiting bacterial biofilm formation. [file Image_1.jpg]

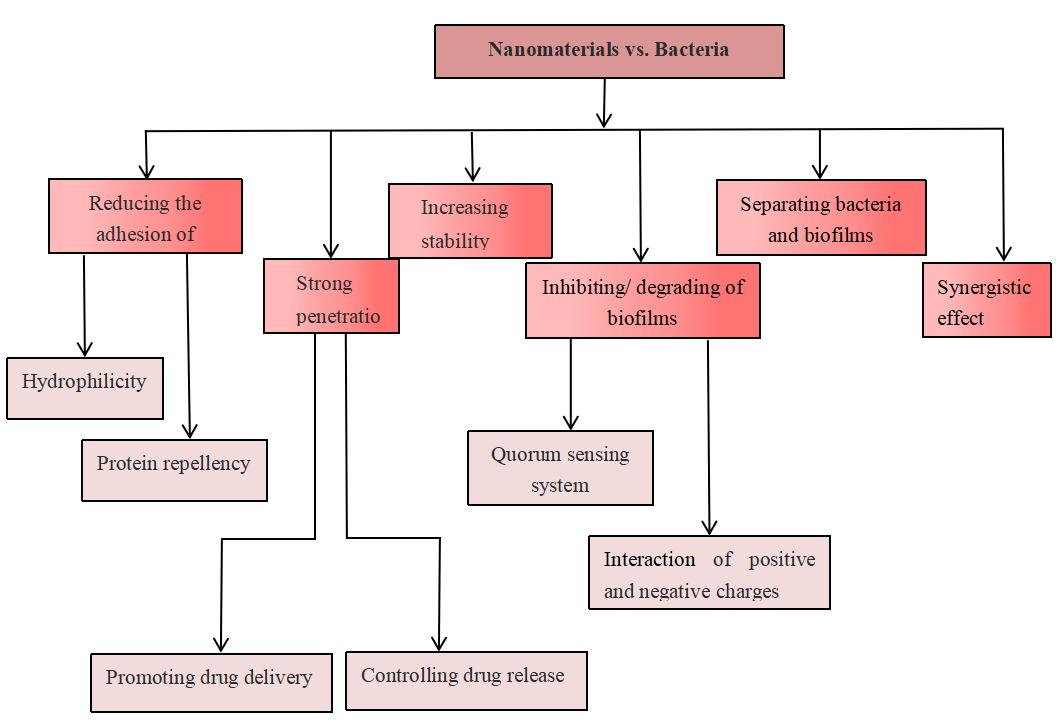

Supplement: Supplementary Figure 2 — Mechanism of action of nanomaterials against biofilms. [file Image_2.jpg]

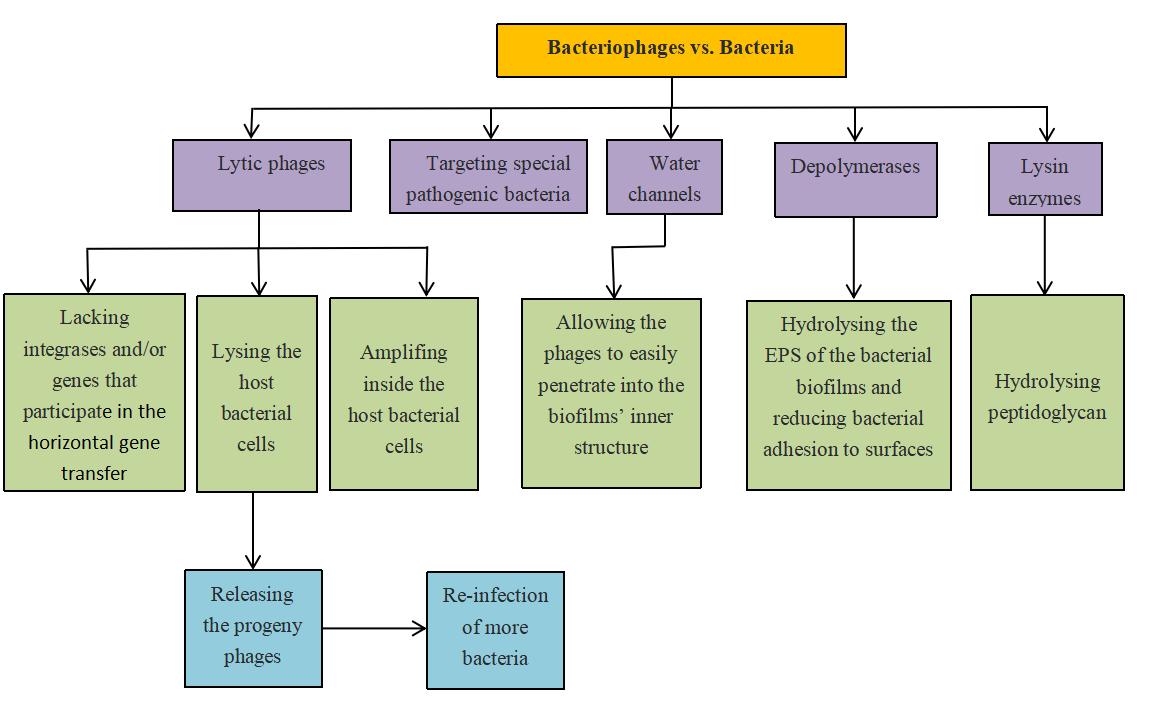

Supplement: Supplementary Figure 3 — Mechanism of action of bacteriophages with anti-biofilm activity. [file Image_3.jpg]

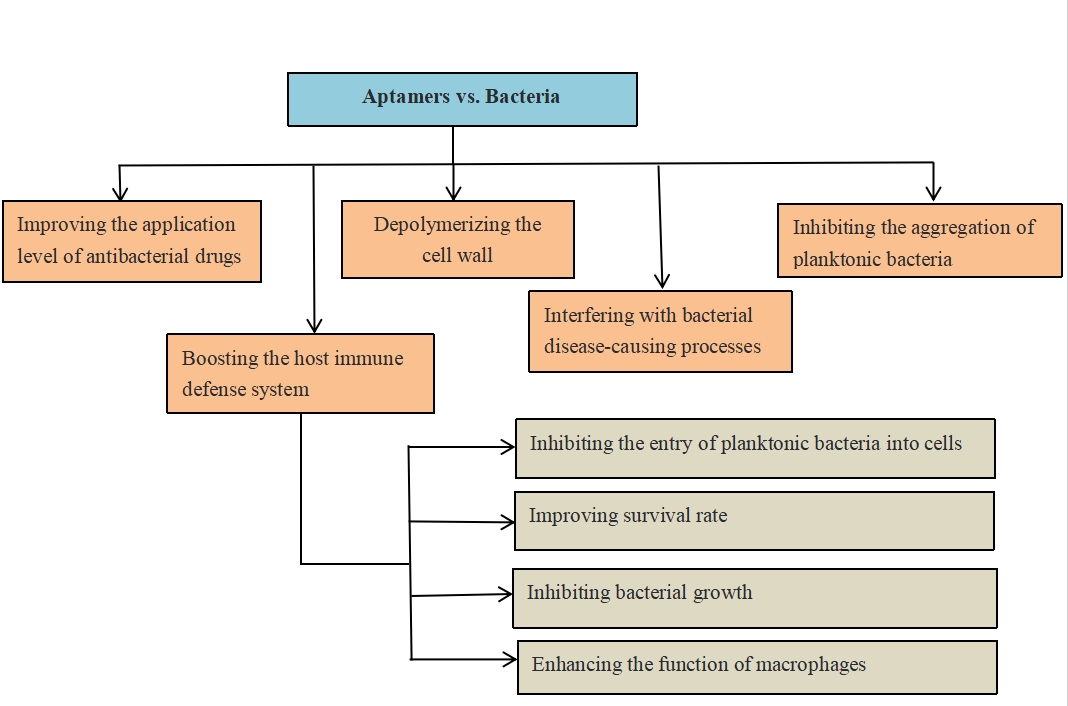

Supplement: Supplementary Figure 4 — Mechanism of action of aptamers with anti-biofilm activity. [file Image_4.jpg]
