# Supplementary material for: Understanding bacterial biofilms: From definition to treatment strategies
Source: Front Cell Infect Microbiol. 2023 Apr 6;13:1137947. doi: 10.3389/fcimb.2023.1137947 (PMC10117668; doi:10.3389/fcimb.2023.1137947)
Supplement: Supplementary Table 1 — Most common bacterial organisms on the surface of medical devices. [file Table_1.doc]

**Supplementary Table 1:** Most common bacterial organisms on the surface of medical devices

| **Implanted Medical Devices** | **Main Microorganisms** | **Authors** |
| --- | --- | --- |
| Cardiovascular Implants | Coagulase-negative *Staphylococcus* spp., *S. aureus* | Kusumoto et al., 2017; Ortega-Loubon et al, 2019 |
| Gastrointestinal Implants  Percutaneous endoscopic gastrostomy  Biliary stenting | *Bacillus*, *Enterococcus*, *Staphylococcus* species  Duodenal microbiota such as *Enterococcus faecium* | Caldara et al., 2022  Vaishnavi et al., 2018 |
| Orthopedic Implants  Prosthetic joint  Osteomyelitis | *S. aureus*, *S.* *epidermidis*, and *Staphylococcus lugdunensis*  *S.* *aureus* | Triffault-Fillit et al., 2019  Lew and Waldvogel. 2004 |
| Neurosurgical Implants | *S. aureus*, *Cutibacterium* spp., *Enterobacter* spp. | Chen et al., 2019 |
| Urological Implants, Stents, and Nephrostomy Tubes | *E. coli*, *P. mirabilis*, *P. aeruginosa*, *E. faecalis*, *Candida tropicalis, Staphylococcus* spp. | Holá et al., 2010 |
| Intravascular Devices | *S. aureus*, *S. epidermis*, *E. faecalis*, *P. aeruginosa*, *K. pneumoniae* | Caldara et al., 2022 |
| Breast Implants | *Staphylococcus* spp., *Streptococcus* spp., *Bacillus* spp., *E. coli*, *Mycobacterium* spp., *Corynebacterium* spp., *Lactobacillus* spp. | Washer and Gutowski. (2012) |
| Dental Implants | Gram-positive cocci, rod-like microbes | Fürst et al., 2007 |
| Microbial keratitis  Contact Lenses | *S. aureus* and *P. aeruginosa*  *P. aeruginosa* | Caldara et al., 2022  Bispo et al., 2015 |
| Intrauterine devices | *S. aureus*, *E. faecalis*, *E. coli*, *Streptococcus* spp., *Actinomyces* spp., *Prevotella* spp., *Bacteroides* spp., *Clostridium* spp., and *C. albicans* | Ádám et al., 2018 |
| Breathing machines | *Streptococcus* spp*.*, *Neisseria* spp., *Prevotella* spp. | Lazarevic et al., 2014 |
| Cochlear implants | *S. aureus*, *C. albicans* | Pawlowski et al., 2005 |

**Supplementary References**

Ádám, A., Pál, Z., Terhes, G., Szűcs, M., Gabay, I. D., and Urbán, E. (2018). Culture- and PCR-based detection of BV associated microbiological profile of the removed IUDs and correlation with the time period of IUD in place and the presence of the symptoms of genital tract infection. Annals of clinical microbiology and antimicrobials, 17(1), 40. doi.org/10.1186/s12941-018-0293-6.

Bispo, P. J., Haas, W., and Gilmore, M. S. (2015). Biofilms in infections of the eye. Pathogens (Basel, Switzerland), 4(1), 111–136. doi.org/10.3390/pathogens4010111.

Chen, Y., Zhang, L., Qin, T., Wang, Z., Li, Y., and Gu, B. (2019). Evaluation of neurosurgical implant infection rates and associated pathogens: evidence from 1118 postoperative infections. Neurosurgical focus, 47(2), E6. doi.org/10.3171/2019.5.FOCUS18582.

Fürst, M. M., Salvi, G. E., Lang, N. P., and Persson, G. R. (2007). Bacterial colonization immediately after installation on oral titanium implants. Clinical oral implants research, 18(4), 501–508. doi.org/10.1111/j.1600-0501.2007.01381.x.

Holá, V., Ruzicka, F., and Horka, M. (2010). Microbial diversity in biofilm infections of the urinary tract with the use of sonication techniques. FEMS immunology and medical microbiology, 59(3), 525–528. doi.org/10.1111/j.1574-695X.2010.00703.x.

Kusumoto, F. M., Schoenfeld, M. H., Wilkoff, B. L., Berul, C. I., Birgersdotter-Green, U. M., Carrillo, R., et al. (2017). 2017 HRS expert consensus statement on cardiovascular implantable electronic device lead management and extraction. Heart rhythm, 14(12), e503–e551. doi.org/10.1016/j.hrthm.2017.09.001.

Lazarevic, V., Gaïa, N., Emonet, S., Girard, M., Renzi, G., Despres, L., et al. (2014). Challenges in the culture-independent analysis of oral and respiratory samples from intubated patients. Frontiers in cellular and infection microbiology, 4, 65. doi.org/10.3389/fcimb.2014.00065.

Lew, D. P., and Waldvogel, F. A. (2004). Osteomyelitis. Lancet (London, England), 364(9431), 369–379. doi.org/10.1016/S0140-6736(04)16727-5.

Ortega-Loubon, C., Muñoz-Moreno, M. F., Andrés-García, I., Álvarez, F. J., Gómez-Sánchez, E., Bustamante-Munguira, J., et al. (2019). Nosocomial Vs. Community-Acquired Infective Endocarditis in Spain: Location, Trends, Clinical Presentation, Etiology, and Survival in the 21st Century. Journal of clinical medicine, 8(10), 1755. doi.org/10.3390/jcm8101755.

Pawlowski, K. S., Wawro, D., and Roland, P. S. (2005). Bacterial biofilm formation on a human cochlear implant. Otology & neurotology : official publication of the American Otological Society, American Neurotology Society [and] European Academy of Otology and Neurotology, 26(5), 972–975. doi.org/10.1097/01.mao.0000169047.38759.8b.

Triffault-Fillit, C., Ferry, T., Laurent, F., Pradat, P., Dupieux, C., Conrad, A., et al. (2019). Microbiologic epidemiology depending on time to occurrence of prosthetic joint infection: a prospective cohort study. Clinical microbiology and infection : the official publication of the European Society of Clinical Microbiology and Infectious Diseases, 25(3), 353–358. doi.org/10.1016/j.cmi.2018.04.035.

Vaishnavi, C., Samanta, J., and Kochhar, R. (2018). Characterization of biofilms in biliary stents and potential factors involved in occlusion. World journal of gastroenterology, 24(1), 112–123. doi.org/10.3748/wjg.v24.i1.112.

Washer, L. L., and Gutowski, K. (2012). Breast implant infections. Infectious disease clinics of North America, 26(1), 111–125. doi.org/10.1016/j.idc.2011.09.003.
