# Supplementary material for: Understanding bacterial biofilms: From definition to treatment strategies
Source: Front Cell Infect Microbiol. 2023 Apr 6;13:1137947. doi: 10.3389/fcimb.2023.1137947 (PMC10117668; doi:10.3389/fcimb.2023.1137947)
Supplement: Supplementary Table 2 — Non-device-related biofilm infections. [file Table_2.doc]

**Supplementary Table 2:** Non-device-related biofilm infections

| **Infection** | **Main Microorganisms** | **Authors** |
| --- | --- | --- |
| Periodontitis | Gram-negative anaerobic bacteria | Mirzaei et al, 2020 |
| Urinary tract infection | Uropathogenic *E. coli*, *E. faecalis*, *K. pneumoniae*, *P. mirabilis*, *Staphylococcus saprophyticus* | Mirzaei et al, 2020 |
| Prostatitis | *E. coli*, *P. aeruginosa, P. mirabilis*, *Serratia* spp., *Enterococcus* spp., *Klebsiella* spp. | Delcaru et al, 2016 |
| Cystic fibrosis | *P. aeruginosa* | Filkins and O’Toole, 2015 |
| Infective endocarditis | *Streptococcus* spp., *Staphylococcus* spp., *Enterococcus* spp. | Elgharably et al, 2016 |
| Otitis media | *H. influenzae*, *S. pneumoniae*, *M. catarrhalis* | Otsuka et al., 2013 |
| Osteomyelitis | *S. aureus*, *Streptococcus* spp., *Enterococcus* spp., *Enterobacteria*, *Mycobacteria*, and *P. aeruginosa* | Brady et al, 2008; Olson and Horswill, 2013 |
| Chronic rhinosinusitis | Over-representation of specific harmful bacterial species (such as *Corynebacterium tuberculostearicum*) and down-representation of other protective microorganisms (*such as Lactobacillus sakei*) | Abreu et al, 2012 |
| Necrotizing fasciitis | Group A *Streptococcus* | Vajjala et al, 2019 |
| Tonsillitis | Group A *Streptococcus* | Mirzaei et al, 2020 |
| Chronic wound | Different bacterial species | Mirzaei et al, 2020 |
| Gastrointestinal infections | *Helicobacter pylori* | Hou et al, 2022 |
| Chronic inflammatory and autoimmune diseases | L-form bacterial pathogens | Marshall and Marshall, 2004 |
| Bacterial vaginosis | *G. vaginalis* and other anaerobes | Li and Zhao, 2020 |

**Supplementary References**

Abreu, N. A., Nagalingam, N. A., Song, Y., Roediger, F. C., Pletcher, S. D., Goldberg, A. N., et al. (2012). Sinus microbiome diversity depletion and *Corynebacterium* tuberculostearicum enrichment mediates rhinosinusitis. Science translational medicine, 4(151), 151ra124. doi.org/10.1126/scitranslmed.3003783.

Brady, R. A., Leid, J. G., Calhoun, J. H., Costerton, J. W., and Shirtliff, M. E. (2008). Osteomyelitis and the role of biofilms in chronic infection. *FEMS immunology and medical microbiology*, *52*(1), 13–22. doi.org/10.1111/j.1574-695X.2007.00357.x.

Li, J., and Zhao, X. (2020). Effects of quorum sensing on the biofilm formation and viable but non-culturable state. Food Res Int. 2020 Nov;137:109742. doi: 10.1016/j.foodres.2020.109742.

Marshall, T. G., and Marshall, F. E. (2004). Sarcoidosis succumbs to antibiotics--implications for autoimmune disease. Autoimmunity reviews, 3(4), 295–300. https://doi.org/10.1016/j.autrev.2003.10.001.

Olson, M. E., and Horswill, A. R. (2013). Staphylococcus aureus osteomyelitis: bad to the bone. *Cell host & microbe*, *13*(6), 629–631. [doi.org/10.1016/j.chom.2013.05.015](https://doi.org/10.1016/j.chom.2013.05.015).

Vajjala, A., Biswas, D., Tay, W. H., Hanski, E., and Kline, K. A. (2019). Streptolysin-induced endoplasmic reticulum stress promotes group A Streptococcal host-associated biofilm formation and necrotising fasciitis. Cellular microbiology, 21(1), e12956. oi.org/10.1111/cmi.12956.
