# Supplementary material for: Understanding bacterial biofilms: From definition to treatment strategies
Source: Front Cell Infect Microbiol. 2023 Apr 6;13:1137947. doi: 10.3389/fcimb.2023.1137947 (PMC10117668; doi:10.3389/fcimb.2023.1137947)
Supplement: Supplementary Table 3 — Microbial and bacterial biofilm-associated infections and their adhesion surfaces. [file Table_3.doc]

Supplementary Table 3: Microbial and bacterial biofilm-associated infections and their adhesion surfaces

| **Microorganism** | **Surfaces** | **Diseases** | **Authors** |
| --- | --- | --- | --- |
| *S. aureus* and *S. epidermidis* | Central venous catheters, heart valves, suture devices, prostheses | Nosocomial infections, endocarditis, mucus cysts, otitis media | Arciola et al., 2012; Qu et al., 2010 |
| *P. aeruginosa* | Contact lenses, central venous catheter, middle ear, prosthesis | Nosocomial infection, cystic fibrosis, otitis media | Wiley et al., 2012; Huse et al., 2013 |
| *S. aureus, E. coli, S. agalactiae* |  | Mastitis | Loera-Muro et al., 2021 |
| *E. coli* | Catheters, ultrasonic instruments, contact lenses, middle ear, prosthesis | Bacterial prostatitis, urinary tract infection, otitis media | Zhang et al., 2019; Khatoon et al., 2018 |
| *S. mutans* | Heart implants, tooth surfaces | Infective endocarditis, dental caries | Metwalli et al., 2013 |
| *E. faecalis* | Heart valves, teeth, central lines | Infective endocarditis, root canal infection | Minardi et al., 2012 |
| 1. *pneumoniae* | Lungs, liver | Respiratory tract infection, pneumonia, lung abscess, liver abscess | Chung, 2016 |
| *H. influenzae* | Middle ear | Otitis media | Takei et al., 2013 |
| *Mycobacterium tuberculosis* | Lungs | Pulmonary tuberculosis | Qvist et al., 2014 |

**Supplementary References**

Chung P. Y. (2016). The emerging problems of Klebsiella pneumoniae infections: carbapenem resistance and biofilm formation. FEMS microbiology letters, 363(20), fnw219. doi.org/10.1093/femsle/fnw219.

Metwalli, K. H., Khan, S. A., Krom, B. P., and Jabra-Rizk, M. A. (2013). Streptococcus mutans, Candida albicans, and the human mouth: a sticky situation. PLoS pathogens, 9(10), e1003616. doi.org/10.1371/journal.ppat.1003616.

Minardi, D., Cirioni, O., Ghiselli, R., Silvestri, C., Mocchegiani, F., Gabrielli, E., et al. (2012). Efficacy of tigecycline and rifampin alone and in combination against Enterococcus faecalis biofilm infection in a rat model of ureteral stent. The Journal of surgical research, 176(1), 1–6. //doi.org/10.1016/j.jss.2011.05.002.

Qvist, T., Johansen, I. S., Pressler, T., Høiby, N., Andersen, A. B., Katzenstein, T. L., et al. (2014). Urine lipoarabinomannan point-of-care testing in patients affected by pulmonary nontuberculous mycobacteria--experiences from the Danish Cystic Fibrosis cohort study. BMC infectious diseases, 14, 655. https://doi.org/10.1186/s12879-014-0655-4.

Takei, S., Hotomi, M., and Yamanaka, N. (2013). Minimal biofilm eradication concentration of antimicrobial agents against nontypeable Haemophilus influenzae isolated from middle ear fluids of intractable acute otitis media. Journal of infection and chemotherapy : official journal of the Japan Society of Chemotherapy, 19(3), 504–509. doi.org/10.1007/s10156-013-0592-y.
