# Supplementary material for: Understanding bacterial biofilms: From definition to treatment strategies
Source: Front Cell Infect Microbiol. 2023 Apr 6;13:1137947. doi: 10.3389/fcimb.2023.1137947 (PMC10117668; doi:10.3389/fcimb.2023.1137947)
Supplement: Supplementary Table 4 — Mechanism of action of antimicrobial peptide. [file Table_4.doc]

Supplementary Table 4: Mechanism of action of antimicrobial peptide

| **Peptide** | **Bacteria** | **Mechanism of action** | **Authors** |
| --- | --- | --- | --- |
| Nisin A | Methicillin-resistant *S. aureus* | Disturbing or destruction of the membrane of biofilm cells | Okuda et al., 2013 |
| Hepcidin 20 | *S. epidermidis* | Reducing the quality of extracellular matrix, targeting PIA to change the structure of biofilm | Brancatisano et al., 2014 |
| Human-defensin 3 | *S. epidermidis* | Reducing the expression of the icaA, icaD, and IcaR genes to decrease biofilm development | Di Somma et al., 2020 |
| IDR-1018 | *S. mutans* | Altering the mRNA expressions of the QS related vicR, vicK, comC and comD genes or block (p)ppGpp | Cao et al., 2020 |
| AMP TAT-RasGAP 317-326 | *A. baumannii*, *P. aeruginosa*, *S. aureus* | Inhibiting the formation and development of biofilms | Heinonen et al., 2021 |
| CRAMP | *P. aeruginosa* | Reducing the c-di-GMP level with a decrease in exopolysaccharides, especially alginate, improving bacterial flagellar motility, increasing the rhamnolipid content | Zhang et al., 2022 |
| Capitellacin | *E. coli* | A low hydrophobicity determines its modest membranotropic activity and slow membrane permeabilization. | Safronova et al., 2022 |
| Cec4 | *A. baumannii* | Affecting multiple metabolic pathways, two-component regulatory systems, quorum sensing and antibiotic synthesis related pathways | Liu et al., 2020 |
| Tilapia piscidin 4 | *G. vaginalis* and *Streptococcus anginosus* | Broad-spectrum antimicrobial and antibiofilm activity | Lin et al., 2022 |
| RP557 | *Mycobacterium* *abscessus* | Down-regulating nitrogen and fatty acid metabolism and peptidoglycan biosynthesis | Li et al., 2022 |
| Spampcin | *P. aeruginosa* | Good thermal stability, certain ion tolerance, and no obvious cytotoxicity | Jiang et al., 2022 |
| Pom-1 and Pom-2 | *C. albicans*, *Candida parapsilosis* | Inhibiting biofilm formation | Raber et al., 2021 |

**Supplementary References**

Brancatisano, F. L., Maisetta, G., Di, Luca. M., Esin, S., Bottai, D., Bizzarri, R., Campa, M., et al. (2014). Inhibitory effect of the human liver-derived antimicrobial peptide hepcidin 20 on biofilms of polysaccharide intercellular adhesin (PIA)-positive and PIA-negative strains of Staphylococcus epidermidis. Biofouling. 2014;30(4):435-46. doi: 10.1080/08927014.2014.888062.

Cao, Y., Yin, H., Wang, W., Pei, P., Wang, Y., Wang, X., et al. (2020). Killing Streptococcus mutans in mature biofilm with a combination of antimicrobial and antibiofilm peptides. Amino Acids. 2020 Jan;52(1):1-14. doi: 10.1007/s00726-019-02804-4.

Di Somma, A., Moretta, A., Canè, C., Cirillo, A., and Duilio, A. (2020). Antimicrobial and Antibiofilm Peptides. Biomolecules. 2020 Apr 23;10(4):652. doi: 10.3390/biom10040652.

Jiang, M., Chen, R., Zhang, J., Chen, F., and Wang, K. J. (2022). A Novel Antimicrobial Peptide Spampcin56-86 from Scylla paramamosain Exerting Rapid Bactericidal and Anti-Biofilm Activity In Vitro and Anti-Infection In Vivo. Int J Mol Sci. 2022 Nov 1;23(21):13316. doi: 10.3390/ijms232113316.

Li, B., Zhang, Y., Guo, Q., He, S., Fan, J., Xu, L., et al. (2022). Antibacterial peptide RP557 increases the antibiotic sensitivity of Mycobacterium abscessus by inhibiting biofilm formation. Sci Total Environ. 2022 Feb 10;807(Pt 3):151855. doi: 10.1016/j.scitotenv.2021.151855.

Liu, W., Wu, Z., Mao, C., Guo, G., Zeng, Z., Fei, Y., et al. (2020). Antimicrobial Peptide Cec4 Eradicates the Bacteria of Clinical Carbapenem-Resistant Acinetobacter baumannii Biofilm. Front Microbiol. 2020 Aug 11;11:1532. doi: 10.3389/fmicb.2020.01532.

Okuda, K., Zendo, T., Sugimoto, S., Iwase, T., Tajima, A., Yamada, S., et al. (2013). Effects of bacteriocins on methicillin-resistant *Staphylococcus aureus* biofilm. Antimicrob Agents Chemother. 2013 Nov;57(11):5572-9. doi: 10.1128/AAC.00888-13.

Raber, H. F., Sejfijaj, J., Kissmann, A. K., Wittgens, A., Gonzalez-Garcia, M., Alba, A., et al. (2021). Antimicrobial Peptides Pom-1 and Pom-2 from Pomacea poeyana Are Active against Candidaauris, C. parapsilosis and C. albicans Biofilms. Pathogens. 2021 Apr 20;10(4):496. doi: 10.3390/pathogens10040496.

Safronova, V. N., Panteleev, P. V., Sukhanov, S. V., Toropygin, I. Y., Bolosov, I. A., and Ovchinnikova, T. V. (2022). Mechanism of Action and Therapeutic Potential of the β-Hairpin Antimicrobial Peptide Capitellacin from the Marine Polychaeta Capitella teleta. Mar Drugs. 2022 Feb 25;20(3):167. doi: 10.3390/md20030167.

Zhang, Y., Cheng, P., Wang, S., Li, X., Peng, L., Fang, R., et al. (2022). *Pseudomonas aeruginosa* biofilm dispersion by the mouse antimicrobial peptide CRAMP. Vet Res. 2022 Oct 8;53(1):80. doi: 10.1186/s13567-022-01097-y.
